# Transcriptional profiling of the developing rat ovary following intrauterine exposure to the endocrine disruptors diethylstilbestrol and ketoconazole

**DOI:** 10.1007/s00204-023-03442-2

**Published:** 2023-01-18

**Authors:** Indusha Kugathas, Hanna K. L. Johansson, Edith Chan Sock Peng, Maryne Toupin, Bertrand Evrard, Thomas A. Darde, Julie Boberg, Monica K. Draskau, Antoine D. Rolland, Séverine Mazaud-Guittot, Frédéric Chalmel, Terje Svingen

**Affiliations:** 1 Univ Rennes, Inserm, EHESP, Irset (Institut de Recherche en Santé, Environnement et Travail) - UMR_S 1085, 9 avenue du Professeur Léon Bernard, 35000 Rennes, France; 2 National Food Institute, Technical University of Denmark, Kemitorvet, Building 202, 2800 Kongens Lyngby, Denmark; 3 SciLicium, 10 rue de la Sauvaie, 35200 Rennes, France

**Keywords:** Endocrine disruptors, Ovary, Puberty, Transcriptomic profiling, BRB-seq, Rat, Risk assessment

## Abstract

Exposure to endocrine-disrupting chemicals (EDCs) during development may cause reproductive disorders in women. Although female reproductive endpoints are assessed in rodent toxicity studies, a concern is that typical endpoints are not sensitive enough to detect chemicals of concern to human health. If so, measured endpoints must be improved or new biomarkers of effects included. Herein, we have characterized the dynamic transcriptional landscape of developing rat ovaries exposed to two well-known EDCs, diethylstilbestrol (DES) and ketoconazole (KTZ), by 3’ RNA sequencing. Rats were orally exposed from day 7 of gestation until birth, and from postnatal day 1 until days 6, 14 or 22. Three exposure doses for each chemical were used: 3, 6 and 12 µg/kg bw/day of DES; 3, 6, 12 mg/kg bw/day of KTZ. The transcriptome changed dynamically during perinatal development in control ovaries, with 1137 differentially expressed genes (DEGs) partitioned into 3 broad expression patterns. A cross-species deconvolution strategy based on a mouse ovary developmental cell atlas was used to map any changes to ovarian cellularity across the perinatal period to allow for characterization of actual changes to gene transcript levels. A total of 184 DEGs were observed across dose groups and developmental stages in DES-exposed ovaries, and 111 DEGs in KTZ-exposed ovaries across dose groups and developmental stages. Based on our analyses, we have identified new candidate biomarkers for female reproductive toxicity induced by EDC, including *Kcne2*, *Calb2* and *Insl3*.

**Supplementary Information:**

The online version contains supplementary material available at 10.1007/s00204-023-03442-2.

## Introduction

Early life exposure to endocrine-disrupting chemicals (EDCs) can affect reproductive development and cause disease later in life. This holds true for both male and female reproductive health (Johansson et al. 2017; Skakkebaek 2017), although the evidence for a causal relationship is much stronger for male reproductive disorders. There is, however, increasing evidence to suggest that female reproductive health is more sensitive to EDCs than previously thought (Buck Louis et al. 2011; Johansson et al. 2017), especially within hormone-sensitive developmental stages during perinatal life; in mice and rats after birth (Johansson et al. 2021). Thus, it is critical to include these windows of development in chemical toxicity testing aiming to detect potential endocrine disruption in females. It is also critical that the tests that are used are sensitive enough for female reproductive toxicity, which may currently not be the case.

Since rats are often used for in vivo reproductive toxicity testing of chemicals, it is important to consider differences in reproductive development between rats and humans. There are obvious temporal differences in ovarian development between the species, which also means that there are differences in susceptible windows of chemical exposure (Johansson et al. 2017). With regard to chemical safety assessments, a prevailing challenge is that current rodent test guidelines may not be sensitive enough to reveal endocrine-disrupting effects that could pose a risk to women’s reproductive health (Draskau et al. 2021; Johansson et al. 2021; OECD 2018). This is not necessarily because there is a lack of effects, but could instead be that the endpoints being assessed are not sensitive or specific enough for their intended purposes (Johansson et al. 2021). This, coupled with the fact that chemical testing and regulation regimens are aiming at rapidly reducing the use of animal testing for chemical safety assessment, means that we must identify appropriate mechanisms of action in order to employ the correct panel of tests in the future.

Diethylstilbestrol (DES) and ketoconazole (KTZ) are well-characterized EDCs. In humans, prenatal exposure to the synthetic estrogen DES increases the incidence of reproductive tract cancers, impairs fertility and causes early menopause in daughters of mothers who have taken DES during pregnancy (Hatch et al. 2006; Hoover et al. 2011; Johansson et al. 2021; Laronda et al. 2012; Palmer et al. 2001; Steiner et al. 2010). KTZ, a pharmaceutical used to treat fungal infections, can perturb steroid hormone synthesis in humans and rodents by inhibiting various cytochrome P450 (CYP) enzymes of the steroidogenesis pathway (Kjærstad et al. 2010; Mason et al. 1987; Munkboel et al. 2019), potentially interfering with both androgen and estrogen synthesis and downstream signaling. KTZ has also been used to reduce the rate of folliculogenesis in women during fertility treatment (Gal et al. 1999; Parsanezhad et al. 2003). However, the mechanisms by which either DES or KTZ cause these adverse female reproductive effects are poorly understood.

We recently performed an in vivo rat reproductive toxicity study to address the lack of sensitive markers for female reproductive toxicity (Johansson et al. 2021). Both DES and KTZ were used to answer some of the questions raised above. To complement and expand on this work, we used the Bulk RNA Barcoding-sequencing (BRB-seq) technology on rat developing ovaries to study the impact of perinatal exposure to DES and KTZ at the transcriptional level and to potentially identify biomarkers of exposure which might be used in future chemical testing strategies. We also performed a cross-species deconvolution-based analysis by combining the BRB-seq dataset with a mouse ovary developmental cell atlas composed of three single-cell RNA sequencing datasets. This was done to validate if the transcriptional changes we observed were indeed related to transcriptional variations or simply to changes in the ratio of different cell types that normally occur during ovary development.

## Materials and methods

### Chemicals

Diethylstilbestrol (DES, CAS no. 56-53-1; purity $\ge$ 99%) and Ketoconazole (KTZ, CAS no. 65277-42-1; purity 98%) were purchased from Sigma-Aldrich and BOC Sciences Inc., USA, respectively. Corn oil was purchased from Sigma-Aldrich (cat.no. C8267-2.5 L) and used as control and vehicle. Solutions used for dosing of animals were stored in glass bottles in the dark at room temperature and continuously stirred during the dosing period.

### Animals and dosing

Animal experiments had ethical approval from the Danish Animal Experiments Inspectorate (license number 2015-15-0201-00553) and were overseen by the in-house Animal Welfare committee. All methods were performed in accordance with relevant guidelines and regulations.

The in vivo rat study was previously described in Johansson et al. (2021). Briefly, time-mated Sprague–Dawley rats (Crl:CD(SD)) (Charles River Laboratories, Sulzfeld, Germany) were delivered on gestational day (GD) 3, with the day following overnight mating denoted GD1. Dams were weighed and assigned to treatment groups with similar body weight (bw) distributions on GD4. Animals were housed in standard conditions with 12 h light/dark cycles and fed a standard soy- and alfalfa-free diet based on Altromin 1314 (Altromin GmbH, Germany) along with ad libitum tap water in Bisphenol A-free bottles (Polysulfone 700 ml, 84-ACBT0702SU Tecniplast, Italy). Animals were housed in pairs until GD17, thereafter individually. Rat dams were exposed to DES or KTZ from GD7 until birth. Dams, and offspring following birth, were weighed and gavaged each morning with vehicle control or test substances.

**Fig.1:**
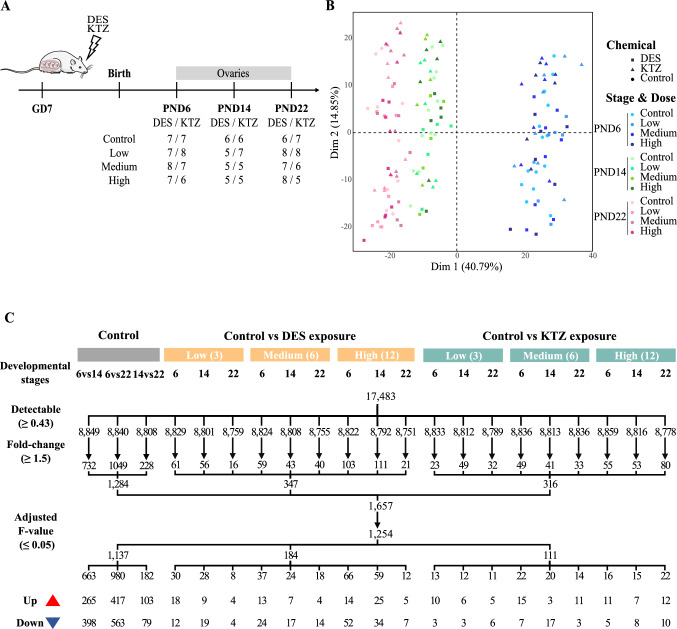
Experimental design, principal component analysis and statistical filtration. **A** Rat dams were orally exposed from gestational day (GD) 7 until birth day, then the offsprings until postnatal day (PND) 6, 14 or 22 via mother’s milk. Exposure doses were 3 (low), 6 (medium) and 12 (high) µg/kg bw/day of DES and 3 (low), 6 (medium) and 12 (high) mg/kg bw/day of KTZ. A total of 156 rat ovaries were collected and sequenced 79 from DES exposure groups and 77 from KTZ exposure groups. **B** Projection on a two-dimension PCA-based space of preprocessed sample data. Ovaries exposed to DES are represented with squares and those exposed to KTZ with triangles. Three colors differentiate the three different stages, PND6 in blue, PND14 in green and PND22 in pink. A color gradient differentiates the doses within each group; lightest for control group and darkest for highest dose group. The first dimension (Dim 1) explains around 41% of variance, discriminating samples according to postnatal days. **C** Differentially expressed genes (DEGs) during rat ovary development and after exposures, analyzed by BRB-seq by comparing controls at PND6, PND14 and PND22 and controls vs low, medium, high doses of DES (3, 6 and 12 µg/kg bw/day) or KTZ (3, 6 and 12 mg/kg bw/day). A total of 1254 DEGs were detected by applying 3 filtration steps for each comparison: (i) the detectable cutoff (0.43); (ii) the fold-change cutoff (1.5) and (iii) the adjusted *F* value (0.05). These genes were then separated into 1137 DEGs during ovary development, 184 DEGs after exposure to DES and 111 DEGs after exposure to KTZ. The number of DEGs found in each condition is indicated in the next row, and the two following rows representing the number of over- or under-expressed DEGs for each condition. Diethylstilbestrol (*DES*), ketoconazole (*KTZ*), bodyweight (*bw*). (color figure online)

Impact of KTZ and DES exposures were studied at three distinct postnatal days (PND 6, 14, and 22) using three different doses of 3, 6 and 12 µg/kg bw/day for DES and of 3, 6 and 12 mg/kg bw/day for KTZ. Ovaries from a total of 159 rats were collected for analysis, giving 5–8 replicates for each experimental condition (Fig. 1A).

### RNA extraction

Total RNA was extracted using an RNA/DNA extraction kit (Qiagen, Germany) following manufacturer’s instructions. For PND6 samples, two ovaries from two littermates were pooled for RNA extraction. For PND14 and PND22, only one ovary from one littermate was used for RNA extraction. RNA quantity was assessed using a NanoDrop^™^ 8000 Spectrophotometer (Thermo Fisher Scientific), and RNA quality using a 2100 Bioanalyzer Instrument (Agilent Technologies, CA, USA) according to manufacturer’s instructions. Only samples with an RNA integrity number (RIN)-score > 7 were included for sequencing.

### BRB-seq library preparation and sequencing

The 3’ Bulk RNA Barcoding and sequencing (BRB-seq) (Alpern et al. 2019) experiments were performed as previously described (Draskau et al. 2021; Giacosa et al. 2021). Briefly, RNAs from ovaries for all treatment groups were distributed onto two 96-well plates, referred to as plate #1 and plate #2. A first step of reverse transcription and template switching reactions was performed using 4 µL total RNA at 2.5 ng/µL and sample-specific barcoded oligo-dT. Subsequently, cDNAs from each plate were pooled, purified and double-strand (ds) cDNAs were synthesized by PCR. The two corresponding sequencing libraries were next built by tagmentation using 50 ng of ds cDNA with the Illumina Nextera XT Kit (Illumina, #FC-131-1024) following the manufacturer’s recommendations. The two resulting libraries were finally sequenced on a NovaSeq sequencer as Paired-End 100 base reads, following Illumina’s instructions by the IntegraGen Company (https://integragen.com/fr/). Image analysis and base calling were performed using RTA 2.7.7 and bcl2fastq 2.17.1.14. Adapter dimer reads were removed using DimerRemover (https://sourceforge.net/projects/dimerremover/).

### Data preprocessing and quality control

Briefly, the first reads (R1) contained 16 bases that were required to have a phred quality score higher than 10. Among these, the first 6 bases corresponded to the unique sample-specific barcode that was needed to de-multiplex the sequencing data, while the following 10 bases corresponded to a unique molecular identifier (UMI) that was used for quantification. The second reads (R2) were aligned to the rat reference transcriptome from the UCSC website (release rn6, downloaded in August 2020) using BWA version 0.7.4.4 with the parameter “−l 24”. Reads mapping to several positions in the genome were filtered out from the analysis. After quality control and data preprocessing, a gene count matrix was generated by counting the number of unique UMIs associated with each gene in lines for each sample in columns. The UMI matrix was further normalized with the regularized log (rlog) transformation package implemented in the DeSeq2 package (Love et al. 2014) (Fig. S1A). Raw and preprocessed data were deposited at the GEO repository under the accession number GSE208545 (Edgar et al. 2002). After quality controls, the number of samples was reduced to 156.

### Differential gene expression analysis

Principal component analysis (PCA) was performed with the FactoMineR (Lê et al. 2008) package implemented in R v4.0.3. Differentially expressed genes (DEGs) were identified based on the following statistical comparisons: (i) PND6 controls vs PND14 controls vs PND22 controls; (ii) controls vs DES-exposed samples at PND6, 14 and 22 and at each dose; (iii) controls vs KTZ-exposed samples at PND6, 14 and 22 at each dose. To avoid potential batch effects between different plates, the comparison of controls at distinct developmental stages was performed using samples from plate #1 only, as these included more replicates. The comparison of KTZ- and DES-exposed samples with their respective controls was performed using plate #1 and plate #2, respectively.

For each comparison, sequential filtration steps (Fig. S1A) were applied: (i) the median gene expression value of all the samples was used as a background cutoff; (ii) a fold-change cutoff of at least 1.5; and, (iii) a statistical filtration with the Linear Models for MicroArray data (LIMMA) package (Smyth 2004) and a *p* value cutoff of 0.05 adjusted with the Benjamini and Hochberg method (Benjamini and Hochberg 1995). Finally, heatmap representations of DEGs were generated with the R package pheatmap. Spot plots were generated with the FlexDotPlot package (Leonard et al. 2022).

The resulting transcriptomic signatures were deposited at the TOXsIgN repository under the accession number TSP1269 (https://toxsign.genouest.org/) (Darde et al. 2018).

### Clustering and functional analysis

The resulting lists of DEGs were clustered into distinct gene expression patterns with the K-means algorithms. A Gene Ontology term enrichment analysis was performed with the Annotation Mapping Expression and Network (AMEN) suite (Chalmel and Primig 2008). A specific annotation term was considered significantly enriched in a given gene expression pattern when the False Discovery Rate (FDR)-adjusted *p* value (Fisher’s exact probability) was ≤ 0.05 and the number of associated genes was $\ge$ 2. KEGG pathways were visualized using the pathview function implemented in R (Luo and Brouwer 2013).

### Assembly of a mouse ovary developmental cell atlas

The analysis of three single-cell RNA sequencing (scRNA-seq) datasets (Long et al. 2022; Meinsohn et al. 2021; Wang et al. 2020) was performed using the Seurat v4.0.1 (Hao et al. 2021) package in R (Fig. S1B). Doublets were filtered out independently in each individual sample using the DoubletFinder R package v.2.0.2 (McGinnis et al. 2019). Cells with less than 1000 UMI, 500 genes and more than 15% of mitochondrial content were removed.

Data for each individual dataset were normalized using the NormalizeData and the SCTransform (by regressing out for the mitochondrial, ribosomal and cell cycle genes) functions implemented into Seurat. The three datasets were then integrated using the RPCA function implemented into Seurat. A principal component analysis (PCA) was performed with the RunPCA function based on the top-3000 most varying genes by excluding mitochondrial, ribosomal and cell cycle genes. Next, cells were projected into a two-dimensional space with the Uniform Manifold Approximation and Projection (UMAP) method implemented into the RunUMAP function based on the top-50 principal components. Cells were then clustered with the FindNeighbors and FindClusters functions with default parameters. Each cell cluster was associated with ovarian cell types based on known marker genes complemented by markers identified by the R package Presto (Korsunsky et al. 2019). A total of 10 broad cell types corresponding to 36 clusters were identified.

### Deconvolution analysis

**Fig.2:**
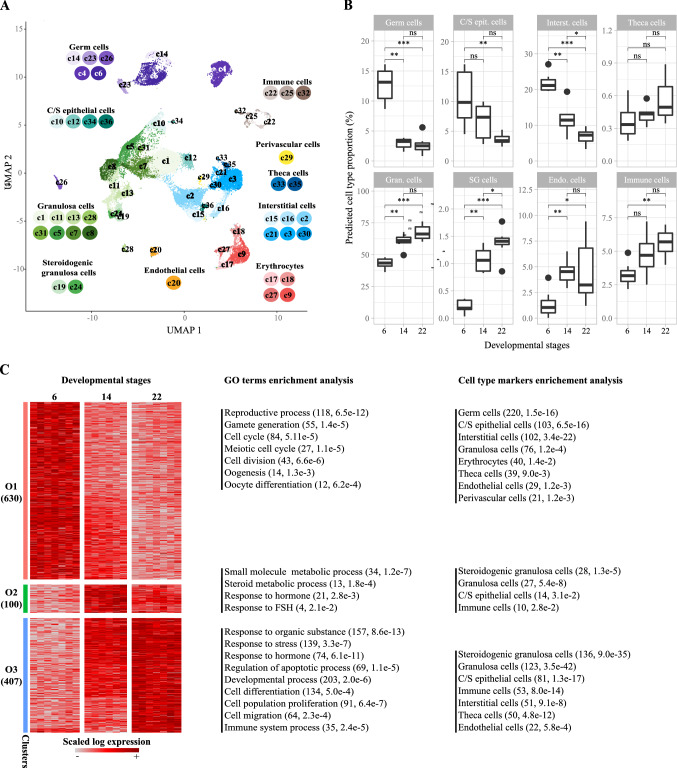
Transcriptional characterization of perinatal ovary development. **A** Uniform manifold approximation and projection (UMAP) representation of single-cell atlas of the developing mouse ovary comprising 35,040 cells divided into 36 cell clusters and showing 10 broad ovarian cell types, including germ cells, immune cells, coelomic/surface (C/S) epithelial cells, theca cells, perivascular cells, interstitial cells, erythrocytes, endothelial cells, granulosa cells and steroidogenic granulosa cells. **B** Boxplot showing predicted cell proportions of the eight most prominent ovarian cell types after deconvolution using single-cell data. Control samples were from the plate containing KTZ-exposed samples (Plate #1). Statistical comparisons were performed using the Wilcoxon rank-sum test, with asterisks indicating statistical significance. *ns* = non-significant; *p ≤ 0.05; **p ≤ 0.01; ***p ≤ 0.001. Coelomic/surface epithelial cells (C/S epit. cells), interstitial cells (Interst. cells), granulosa cells (Gran. cells), steroidogenic granulosa cells (SG cells), endothelial cells (Endo. cells). **C** Heatmap representation of DEGs during rat ovary development revealed by BRB-seq through comparisons of control ovaries at PND6, PND14 and PND22. In total, 1137 DEGs were detected and grouped into 3 expression patterns (O1–O3) underpinning rat ovary development. Each row represents a gene and each column a time point. The color code indicates scaled gene expression values. GO terms enrichment analysis revealed biological processes terms significantly associated with each expression pattern, which are indicated on the right with associated number of genes and *p* value. Besides, enriched cell types based on corresponding markers are provided for each pattern.

The MuSiC R package was used for the deconvolution analysis (https://github.com/xuranw/MuSiC) (Wang et al. 2019). MuSiC is a method that utilizes cell type-specific gene expression from scRNA-seq data to characterize cell type compositions from bulk RNA-seq data in complex tissues (Wang et al. 2019). For the subsequent analysis, each bulk sample was deconvoluted to estimate the proportion of each individual cell type described in the mouse ovary developmental cell atlas. Briefly, count matrices of scRNA-seq data and BRB-seq data were used as input data. To assist the deconvolution algorithm with cell type-specific genes conserved across mice and rats, the scRNA-seq matrix was reduced to only variable genes with unique mouse-to-rat ortholog, i.e., one-to-one relationship as described in the set of homologs available on Ensembl v105 (Howe et al. 2021). The deconvolution analysis was performed on each cell cluster described in the mouse ovary developmental cell atlas (Fig. 2A). The proportions of ten broad cell types (including coelomic/surface epithelial cells, interstitial cells, theca cell, granulosa cells, steroidogenic granulosa cells, germ cells, endothelial cells, immune cells, erythrocytes and perivascular cells) were estimated by summing the predicted proportions of their associated cell clusters. Statistical comparisons were performed using the Wilcoxon rank-sum test.

## Results

### A cross-species deconvolution-based strategy confirms significant changes in ovarian cellularity during perinatal development

To further characterize toxicological effects in the developing rat ovaries exposed to DES and KTZ (Johansson et al. 2021), we sought to analyze potential transcriptional changes using the BRB-seq technology. Total RNA from 156 postnatal ovaries from 12 experimental groups were sequenced, comprising 3 dose groups each of DES- and KTZ-exposed rats with respective control groups, at 3 distinct developmental stages: PND6, PND14 and PND22 (Fig. 1A). Following data normalization, the 156 transcriptomes were projected on a 2D PCA-based space whose first component explaining 41% of the variation (x-axis) clearly differentiated samples according to the three developmental stages (Fig. 1B). Notably, the two first components of the PCA analysis could not discriminate between samples based on exposure scenarios.

We next performed a cross-species deconvolution analysis to predict the relative proportion of distinct ovarian cell types in each BRB-seq sample. Since rat scRNA-seq datasets describing the developing ovary were not available at the time of the analysis, we instead assembled a mouse ovary developmental cell atlas. This atlas was composed of nine samples ranging from embryonic day 16.5 (E16.5) to adulthood by integrating control samples from three studies (Long et al. 2022; Meinsohn et al. 2021; Wang et al. 2020). After quality controls, the number of detected cells per sample ranged from 610 to 16,202 (Fig. S2A). The median number of detected genes per cell ranged from 917 to 5738. The final scRNA-seq dataset contained 35,040 cells that were projected on a 2D space (UMAP) (Fig. S1B). Cells were next partitioned into 36 cell clusters (termed c1–c36) (Fig. S2B) that were associated with ten broad cell types based on specific marker genes (Fig. S2, panels C–D, Table S3).

Germ cells (associated with five cell clusters including c4, c6, c14, c23, and c26) were identified based on the expression of *Ddx4*, *Dazl*, *Sycp3* and *Kit* (Fig. S2 C, D). Four clusters (c10, c12, c34, and c36) were classified as coelomic or surface epithelial cells as they expressed canonical markers such as *Upk3b* and *Krt19*. Height clusters (c1, c5, c7, c8, c11, c13, c28, and c31) were associated with granulosa cells and two others (c19, c24) with steroidogenic granulosa cells based on the expression of *Fst*, *Foxl2*, *Nr5a1*, *Wnt6*, and *Runx1* as well as genes encoding steroidogenic enzymes (such as *Cyp17a1*, *Cyp19a1*, *Cyp11a1*, *Star*, and *Hsd3b1*). Eight clusters (c2, c3, c16, c15, c21, c30, c33, and c35) were associated with interstitial cells among which two corresponded to theca cells (c33, c35) based on the expression of *Pdgfra*, *Tcf21*, *Dcn* and *Ptch1*. The remaining clusters were classified as endothelial cells based on *Cd34* and *Pecam1* (c20), perivascular cells based on *Pdgfrb* and *Acta2* (c29), erythrocytes based on *Snca* and *Alas2* (c9, c17, c18, and c27), and finally immune cells based on *Ptprc* (c22, c25, and c32).

With the exception of a difference in the germ cell population at PND14, no significant difference in overall cell numbers were observed between the two BRB-seq plates (#1 and #2), which reflects good reproducibility within the study (Fig. 2B, Fig. S3). When comparing cell populations in control ovaries from plate #1 across developmental stages, a shift in overall cell type proportion for almost all cell populations was detected: (i) a decrease in proportion of germ cells (− 81%, 5.8e-4); (ii) a decrease in proportion of coelomic/surface epithelial cells (− 65%, 4.1e−3) and interstitial cells (− 66%, 5.8e−4) concomitant with, (iii) an increase in the proportion of granulosa (+ 53%, 5.8e−4), steroidogenic granulosa (+ 600%, 5.8e−4) and theca cells (+ 67%, 7.3e−2); and finally, (iv) variations in immune (+ 78%, 2.3e−3) and endothelial (+ 340%, 4.7e−3) cell populations (Fig. 2B).

### Temporal changes in the rat ovary transcriptome between PND6 and PND22

Statistical comparisons of control ovaries at distinct developmental stages, or control samples to either DES- or KTZ-exposed samples, identified 1254 significantly differentially expressed genes (DEGs) (Fig. 1C, Table S1) among which 1137 were differentially expressed during ovarian development(Figs. 1C, 2C, Table S1). DEGs related to the developing ovary were subsequently clustered into three expression patterns (named O1–O3) showing transcriptional transitions across developmental stages (Fig. 2C). The functional analysis of those expression patterns highlighted 479 enriched biological processes and 22 pathways (Table S2, Fig. 2C). Briefly, the O1 expression pattern displayed highest expression at PND6 and was significantly enriched in genes associated with ‘reproductive process’ (118 genes, adjusted *p* value 6.5e−12), gamete generation (55, 1.4e−5), ‘oogenesis’ (14, 1.3e−3) and ‘oocyte differentiation’ (12, 6.2e−4), as well as ‘cell cycle’ (84 genes, adjusted *p* value 5.1e−5), ‘cell division’ (43, 6.6e−6) and ‘meiotic cell cycle’ (27, 1.1e−5). As expected, O1 included well-known oocyte/germline-related genes such as *Ddx4*, *Sycp1*, *Syce1*, *Syce2*, *Hsf2bp*, *Dazl*, *Lhx8* and *Nlrp5* (Table S2). Functional analysis based on markers identified from scRNA-seq data demonstrated O1 to be significantly enriched for markers of germ cells (220, 1.5e−16), coelomic/surface epithelial cells (103, 6.5e−16), interstitial cells (102, 3.4e−22), granulosa cells (76, 1.2e−4), erythrocytes (40, 1.4e−2), theca cells (39, 9.0e−3), endothelial cells (29, 1.2e−3) and perivascular cells (21, 1.2e−3). Genes belonging to O2 were most highly expressed at PND14, were significantly associated with terms such as ‘response to hormone’ (21, 2.8e−3) or ‘small molecule metabolic process’ (34, 1.2e−7) and were enriched in marker genes for steroidogenic granulosa cells (28, 1.3e−5), granulosa cells (27, 5.4e−8), coelomic/surface epithelial cells (14, 3.1e−2) and immune cells (10, 2.8e−2). Finally, the O3 expression pattern showed highest expression at PND22 and was enriched in biological processes related to ‘response to stress’ (139, 3.3e−7), ‘regulation of apoptotic process’ (69, 1.1e−5), ‘developmental process’ (203, 2.0e−6), ‘cell differentiation’ (134, 5.0e−4), and ‘cell migration’ (64, 2.3e−4). It was enriched with markers for steroidogenic granulosa cells (136, 9.0e−35), granulosa cells (123, 3.5e−42), coelomic/surface epithelial cells (81, 1.3e−17), immune cells (53, 8.0e−14), interstitial cells (51, 9.1e−8), theca cells (50, 4.8e−12) and endothelial cells (22, 5.8e−4) (Table S2, Fig. 2C).

### DES exposure induces significant transcriptional changes in the developing rat ovary

The cross-species deconvolution analysis was performed on the DES transcriptomic data (plate #2) to estimate whether DES exposure could induce changes to ovary cellularity. The relative proportion of each cell type was statistically compared between DES samples and their corresponding control samples at each exposure dose (low = 3 µg/kg bw/day; medium = 6; high = 12) and at each developmental stage (PND6, PND14 and PND22) (Fig. S4). With the exception of a decrease of coelomic/surface epithelial cells (−46%, 1.7e−2) and a slight increase of granulosa cells (+ 17%, 3.8e−2) at PND6 in the highest dose, this analysis did not reveal any other significant changes.

**Fig. 3:**
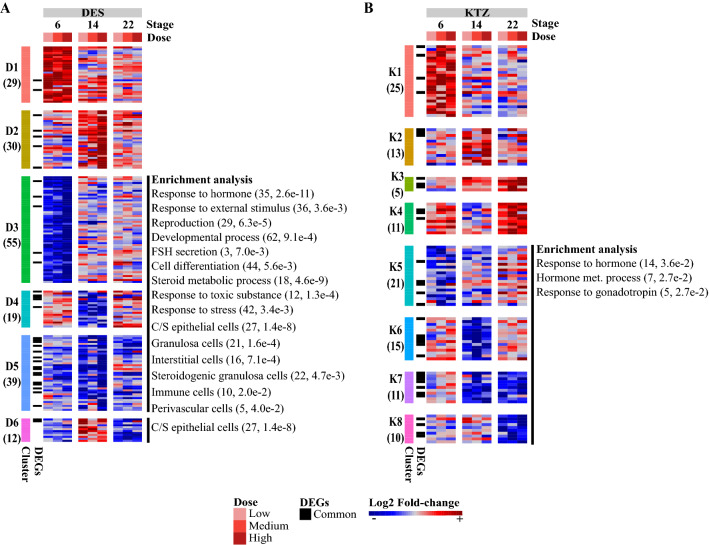
Heatmap representation of differentially expressed genes (DEGs) induced by DES and KTZ. **A** DES exposure induced 184 DEGs in the ovaries at postnatal days 6, 14 and 22 at 3 different exposure doses (3, 6 and 12 µg/kg bw/day), and were grouped into 6 expression patterns (D1–D6). **B** 111 DEGs induced by KTZ exposure induced 111 DEGs at postnatal days 6, 14 and 22 at 3 different exposure doses (3, 6 and 12 mg/kg bw/day) and were grouped into 8 expression patterns (K1–K8). Each row represents a gene and each column a combination of time and dose. The color scale indicates scaled log2 fold-change values. The matching biological processes and cell types are shown on the right, with the associated number of genes and *p* value. DEGs that are common between DES and KTZ exposure are indicated in black on the left of the heatmap.

The pairwise comparisons between ovaries exposed to DES and control samples at each developmental stage, and at each dose, identified 184 DEGs (Fig. 1C, Fig. 3A, Table S1) of which 62, 71 and 128 showed significant changes at the low, medium and high doses, respectively. Conversely, out of the 184 DEGs, 91, 85 and 27 genes showed significant changes at PND6, PND14 and PND22, respectively. The resulting DEGs were further clustered into six expression patterns (named D1–6) which were subjected to a functional analysis (Fig. 3A, Table S2). Patterns D1 and D2 comprised 59 genes that were over-expressed following DES exposure, but not enriched in specific biological processes. Patterns D3, D4 and D5 comprised 113 genes that were under-expressed after DES exposure. These DEGs were significantly associated with 178 biological processes and six pathways (Fig. 3A, Table S2), including ‘response to stress’ (42, 3.4e−3), ‘response to external stimulus’ (36, 3.6e−3),’response to hormone’ (35, 2.6e−11) and ‘developmental process’ (62, 9.1e−4). Pattern D6 was composed of genes with a mixed expression pattern; i.e., showing overexpression at PND14 and underexpression at PND22, but were not enriched in specific biological processes. In clusters D1 and D3 at PND6, some gene transcripts stood out with interesting expression profiles specific to DES exposure. In cluster D1, expression of *Dusp10* and *Homer2* were upregulated in all dose groups, and *Tex101* in two dose groups (Fig. 3A, Table S1). In cluster D3, *Rspo1* expression was decreased in all dose groups, whereas *Sycp3* and *Wt1* were downregulated in two dose groups (Fig. 3A, Table S1). These genes could be potential candidate biomarkers for estrogen-like activities. The functional analysis based on single-cell, cell-specific markers revealed that the under-expressed patterns (D3–5) were significantly enriched for markers of coelomic/surface epithelial cells (27, 1.4e−8), granulosa cells (21, 1.6e−4), interstitial cells (16, 7.1e−4), steroidogenic granulosa cells (22, 4.7e−3), immune cells (10, 2.0e−2) and perivascular cells (5, 4.0 e−2). Together, these results indicate that the decrease of coelomic/surface epithelial cell markers might be due to the slight decrease of the coelomic/surface epithelial cell proportion observed at PND6 with the cross-species deconvolution analysis.

### KTZ exposure induces significant transcriptional changes in the developing rat ovary

With the exception of a decrease in interstitial cells at the lowest dose (−42%, 2.9e−2) and at the highest dose (−58%, 1.8e−2) at PND22, the cross-species deconvolution analysis of the KTZ transcriptomic data (plate #1) did not reveal any other significant variations in relative cell proportions (Fig. S5). The transcriptomic analysis identified 111 DEGs showing an altered expression after exposure to KTZ, including 36, 53 and 49 genes at the low, medium and high doses, respectively (Fig. 1C, Fig. 3B, Table S1). The majority of the transcriptional changes were observed at the two highest exposure doses. Among the 111 DEGs, 43, 40 and 40 genes showed an altered expression level at PND6, PND14 and PND22, respectively. Contrary to DES, transcriptional changes induced by KTZ exposure were not linear dose-responses in the current study. The 111 DEGs were next partitioned into eight expression clusters (termed K1–K8) (Fig. 3B). Clusters K1, K2, K3 and K4 comprised 54 genes over-expressed at PND6, PND14, PND14-22 and PND22, respectively. Among the genes specifically altered after exposure to KTZ, *Hormad1* in K1 and *Sox9* in K4 were consistently over-expressed whatever the dose at PND6, and could therefore be considered as potential candidate biomarkers for exposure to steroidogenic inhibitors. The functional analysis did not reveal any enriched terms. Conversely, expression patterns K5, K6, K7 and K8 included 57 genes under-expressed at PND6, PND14, PND14–22 and PND22, respectively. These DEGs were significantly associated with nine biological processes (Table S2) related to ‘response to hormone’ (14, 3.6e−2), ‘hormone metabolic process’ (7, 2.7e−2) and ‘response to gonadotropin’ (5, 2.7e−2). The cell type functional analysis did not reveal any cell type enrichment.

### Transcriptional signature comparison of DES and KTZ points to candidate biomarkers of exposure

**Fig. 4:**
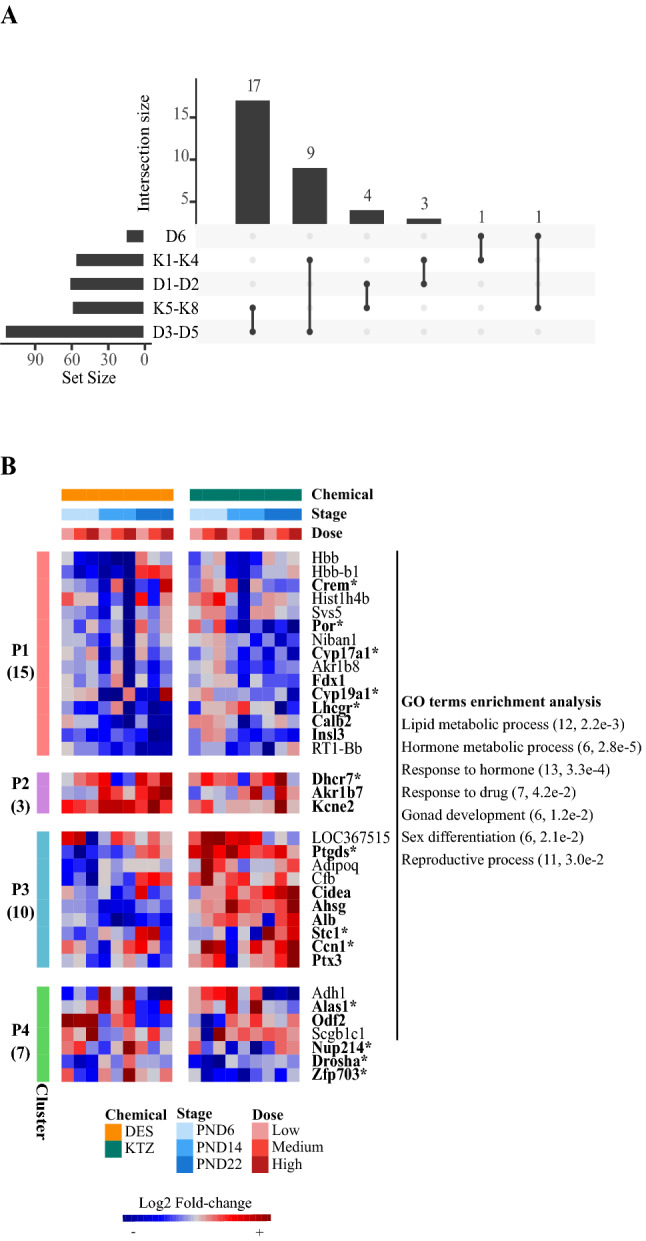
Identification of shared differentially expressed genes (DEGs) induced by DES and KTZ. **A** Intersection plot indicating the number of DEGs shared by DES and KTZ over-expressed (D1–D2 and K1–K4), under-expressed (D3–D5 and K5–K8) or mixed expression patterns (D6). **B** Heatmap representation of the 35 shared DEGs after exposure to DES and KTZ, which were grouped into four expression patterns (P1–P4). The number of DEGs in each cluster is indicated on the left of the heatmap. The matching GO terms are shown on the right with the associated number of genes and *p* value. The color code indicates scaled log2 fold-change values. Genes that are found in the single-cell data are indicated in bold. Those that were also found as cell type markers are indicated with asterisks

We next sought to identify potential biomarkers for sensitive, reliable and reusable endpoints related to female reproductive toxicity that may be used for future chemical hazard identification or safety assessments. The transcriptomic analysis of perinatal ovaries exposed to either DES or KTZ identified 184 and 111 DEGs, respectively. Of these, 35 were affected by both DES and KTZ (Fig. 4A) which were subsequently partitioned into four clusters (termed P1–4) (Fig. 4B, Table S1). Genes belonging to P1 (15 genes) were downregulated after exposure to DES and KTZ. Conversely, cluster P2 (3 genes) included genes that were upregulated. Cluster P3 comprised 10 genes that were predominantly downregulated after exposure to DES, but upregulated after exposure to KTZ. Finally, genes from P4 showed a complex expression pattern for which it is difficult to identify common characteristics. Functional analysis of the 35 shared DEGs identified 64 enriched biological processes (Table S2, Fig. 4B), including ‘lipid metabolic process’ (12, 2.2e−3), ‘hormone metabolic process’ (7, 4.7e−4), ‘response to hormone’ (13, 3.3e−4), ‘gonad development’ (6, 1.2e−2), ‘sex differentiation’ (6, 2.1e−2) or ‘reproductive process’ (11, 2.9e−2). The overwhelming majority of those terms were specifically associated with P1 (Fig. 4B). Pathway analysis revealed that ovarian steroidogenesis was significantly associated with P1 (3, 1.1e−2). Indeed, the expression patterns of the three genes *Cyp17a1*, *Cyp19a1* and *Lhcgr* of the ovarian steroidogenesis pathway were similarly affected by DES and KTZ exposures, whereas the expression of several other genes encoding for steroidogenic enzymes (i.e., *Star*, *Hsd3b3*, *Hsd17b1* and *Akr1c15*) were only altered by one of the two chemicals (Fig. S6). This suggests that expression, and potentially function, of critical steroidogenic enzymes are negatively affected by DES and KTZ exposure during rat ovary development. Among the 35 shared DEGs, 23 were found in the mouse ovary developmental cell atlas, with 13 corresponding to marker genes associated with specific cell type populations (Fig. S7, Table S3). *Crem* and *Stc1* were associated with theca cells, *Por* and *Ptgds* with coelomic/surface epithelial cells, *Cyp17a1*, *Cyp19a1*, *Lhcgr* and *Dhcr7* with steroidogenic granulosa cells, and *Nup214* and *Zfp703* with germ cells. *Ccn1* is associated with coelomic/surface epithelial cells, interstitial cells and perivascular cells, *Drosha* with germ and steroidogenic cells, and finally *Alas1* with immune and granulosa cells. Importantly, three regulated genes belonging to P2 (*Kcne2*, *Dhrc7*, *Akr1b7*) were identified as being upregulated at several doses and developmental stages in both DES and KTZ. We also identified *Insl3* in P1, which tended to be under-expressed whatever the dose of either DES or KTZ. These up- or downregulated genes might be good candidate markers of exposure to investigate in the near future.

## Discussion

With the use of BRB-seq technology, we have analyzed transcriptional changes in the developing rat ovary following exposure to two well-known endocrine disruptors DES and KTZ. These analyses were conducted to complement our previous in vivo toxicity study (Johansson et al. 2021) where we reported on reproductive effect outcomes, but with a specific aim to potentially identify sensitive biomarkers of ovarian dysgenesis.

We first performed a cross-species deconvolution analysis to identify changes to rat ovarian cellularity during early postnatal development. The relative proportion of germ cells was drastically reduced between PND6 and PND22, concomitant with a relative decrease in interstitial cells, and an increase in theca cells. These changes in tissue cellularity correspond with known biological events occurring during ovary development (Picut et al. 2015), for instance the death of the first wave of follicles (McGee et al. 1998) and the occurrence of a second wave of follicle formation by around PND14 (Picut et al. 2015). Despite massive follicular atresia, we also observed a relative increase in the granulosa cell proportion, which could be explained by the continuous recruitment of subsequent waves of follicle growth rather than a bias of the deconvolution-based analyses. Notably, cross-species differences between rats and mice could introduce some limitations with respect to deconvolution using the mouse single-cell dataset; however, our approach allowed for a broad classification of genes consistent with known cell types of both species. In addition, it identified steroidogenic granulosa cells, consistent with known profiles of the first waves of follicle growth in rats (Guigon et al. 2003; Mazaud et al. 2002), mice and humans (François et al. 2017), suggesting that cross-species comparisons was a valid approach in this instance.

With respect to ovaries from rats exposed to either DES or KTZ, we did not observe any significant changes in overall cell type numbers when compared to control ovaries. Thus, with the exception of coelomic/surface epithelial cell numbers that were slightly decreased at PND6 in the DES-exposed ovaries, we surmised that any observed changes to transcript levels in the exposed ovaries were most likely due to bona fide effects on gene expression and not simply a consequence of changes to tissue cellularity. In other words, we have demonstrated that a cross-species deconvolution approach can confirm a good correspondence between mouse and rat developmental windows between PND0 to PND14 (Cardoso-Moreira et al. 2019). Given the critical importance of the rat model in toxicology, however, it would be valuable to assemble a rat ovary developmental cell atlas to further improve such deconvolution-based analyses.

During the course of postnatal ovarian rat development, we found 1137 DEGs divided into 3 expression patterns (O1–3) showing consecutive peak expression at PND6, PND14 and PND22. Each pattern was associated with specific processes including oogenesis and gamete generation in O1 at PND6, response to hormone in O2 and O3 from PND14 onwards, and response to stress and apoptotic process in O3 at PND22. This is consistent with the high relative ratio of oocytes at PND6, the growth of healthy follicles based on a high rate of proliferation of granulosa cells at PND14 with differentiating theca, and with the beginning of follicle atresia at PND22 (Mazaud et al. 2002; Picut et al. 2015). Noteworthy, the PND6-22 window of development encompasses the first waves of follicle growth, which were already shown to be atypical (François et al. 2017; Mazaud et al. 2002). Although not the focus of this study, these developmentally regulated genes display a pattern consistent with what is known about the program underlying ovary development in the rat and thus attest to the robustness of our BRB-seq dataset.

Most DEGs induced by DES exposure were significantly differentially expressed at PND6 (91 DEGs) and PND14 (85 DEGs), and less at PND22 (27 DEGs). This suggests either an age-related effect with more marked effect at earlier stages, or a cell type-related effect, considering the evolution of cell type ratios in the postnatal ovary. Our results also indicate dose-dependent effects of DES, with a higher dose corresponding to a higher number of transcriptional alterations in the developing ovary. In addition, the majority of the DEGs were downregulated after DES exposure (113 out of 184 DEGs). Functional analysis of these revealed a direct response to external stimuli, including response to hormone signaling, stress responses and general cell differentiation. In the absence of gross changes in ovarian cellularity, as shown by deconvolution analyses, these responses could be indicative of disrupted perinatal development. Interestingly, several genes whose expression has already been described as atypical in the development of the first waves of follicular growth in immature rats were deregulated at PND14 (Guigon et al. 2003; Mazaud et al. 2002). Genes displaying lower expression levels in exposed ovaries typically associate with the first wave of follicle growth in granulosa cells (e.g., *Inhbb*, *Cyp19a1*, and *Inhba*) and theca cells (*Star*, *Cyp17a1*, *Cyp11a1*, *Lhcgr*, and *Insl3*). Since this effect peaked at PND14, it suggests that this first follicular wave may have been altered by DES exposure, which would be consistent with almost 50% of the downregulated genes known to be involved in developmental processes. In general, the early effect on the transcriptome by DES is associated with the enrichment in genes involved in development, which is consistent with the in vivo effects observed on immature rat ovaries (Johansson et al. 2017). Thus, potential biomarkers of estrogenic effects, selected at PND6, could include *Homer2*, *Dusp10*, *Tex101*, *Sycp3*, *Wt1* and *Rspo1*, as they were all affected by exposure to DES for at least two doses.

Compared to DES, KTZ induced a comparable low number of transcriptional changes at PND6 (43 DEGs), PND14 (40) and PND22 (40), with only a few genes intersecting between the groups, i.e., *Sox9* and *Ccn1*, suggesting stage-dependent modes of action. Functional analysis revealed dysregulation of genes involved in the response to hormones such as *Cyp17a1*, *Fdx1*, *Por*, *Cyp19a1* and *Lhcgr*. KTZ also had much less effect on genes associated with atypical expression in the first waves of follicular growth in immature rats than what was observed for DES. For example, *Cyp19a1* and *Inhba*, both of which are strongly expressed in the granulosa cells of the first waves, were not significantly affected by KTZ with the exception of reduced expression of *Cyp19a1* at PND22. This may suggest that the first wave of follicular growth is maintained, but may disappear prematurely, or that its steroidogenic function contributing to the E2 spike is affected specifically. Differentiation of theca cells was also affected by KTZ, with under-expression of genes such as *Cyp17a1*, *Fdx1*, *Por*, or *Insl3* from PND14. This suggests an effect on the endocrine function of the first waves of follicle growth rather than the growth itself, since the deconvolution analysis revealed little effects on overall cellularity of the ovaries. Such subtle alterations may not massively modify the maturation of the whole ovarian brain axis, and subsequent puberty regulation, in these KTZ-exposed animals (Johansson et al. 2021), consistent with the absence of impact of disrupted first wave of follicular growth on puberty parameters (Mazaud et al. 2002). Promising potential biomarkers of exposure to steroidogenesis inhibitors were identified at PND6, and include the over-expressed *Hormad1*, which is a critical protein involved in meiosis I checkpoint (Shin et al. 2013) and *Sox9*, which can be found in adult mouse theca interna cells of pre-antral/antral follicles (Notarnicola et al. 2006) and is also found to be affected after exposure to triticonazole (Draskau et al. 2022).

Comparison of both DES and KTZ transcriptomic signatures identified 35 common genes that were further partitioned into four expression patterns (P1–P4). While gene ontology consistently showed an enrichment of genes associated with response to drugs, we also found that most of the common DEGs associated with ovarian steroidogenesis. This suggests that ovarian steroidogenesis is highly sensitive to chemicals in immature rat ovaries, at least at the transcript level. While some genes displayed very distinct transcriptional changes when comparing DES and KTZ exposures (expression patterns P3 and P4), others displayed reasonably similar transcriptional profiles after exposure to DES and KTZ (expression patterns P1 and P2). This makes them promising potential biomarkers of endocrine disruption.

Expression pattern P1 included 15 genes downregulated after exposure to DES and KTZ, and it could thus be interpreted as a loss of cellular function. It includes well-known genes encoding for protein involved in steroid biosynthesis (*Cyp17a1*, *Cyp19a1*, *Fdx1*, *Lhcgr* and *Por*) (Yazawa et al. 2019), gonad development (*Ahsg*, *Insl3*, *Lhcgr*, and *Ptx3*) (Chartrain et al. 1984; Fisher et al. 1998; Mack et al. 2000; Scarchilli et al. 2007; Zhang et al. 2001; Zimmermann et al. 1999), or the calcium signaling factor *Calb2*, which may also be involved in steroidogenesis (Schwaller 2014). *Calb2* has for long been known to be expressed in the ovaries of rodents and humans (Bertschy et al. 1998; Lugli et al. 2003; Pohl et al. 1992), but its function remains unclear. Notably, expression mainly localizes to the androgen-producing theca cells (Bertschy et al. 1998; Pohl et al. 1992) and in rats, there is a surge in *Calb2* at around PND19 corresponding to theca cell recruitment and activation of the hypothalamus–pituitary–gonadal (HPG) axis (Picut et al. 2015). Correspondingly, *Calb2* is expressed by androgen-producing Leydig cells of the testis (Altobelli et al. 2017; Strauss et al. 1994), where it has been suggested to be involved in the regulation of steroidogenesis (Xu et al. 2018). We recently identified *Calb2* as a putative biomarker for female reproductive toxicity by performing a proteomics screen on rat ovaries exposed during development to a mixture of environmental chemicals (Johansson et al. 2020). Subsequently, we have observed dysregulated *Calb2* expression in fetal rat testis exposed to flusilazole (Draskau et al. 2021). Based on these observations, it would be of interest to scrutinize further if *Calb2* could serve as a broad biomarker for gonadal toxicity, especially pertaining to perturbed steroidogenesis and reproductive function.

Expression pattern P2 included *Kcne2*, *Dhcr7* and *Akr1b7* that were all upregulated in several exposure groups (several doses and developmental stages). *Kcne2* encodes an ion transmembrane transport and voltage-gated potassium channel protein (Kundu et al. 2008) involved in cardiac arrhythmia (Abbott 2012; Papanikolaou et al. 2021). While its role during gonad development is not clearly established, several potassium channels are known to participate in the regulation of progesterone secretion (Kim et al. 2020). *Dhcr7* encodes an enzyme involved in the cholesterol biosynthesis (Nakanishi et al. 2021). It has been identified as a biomarker of exposure to KTZ and DES in human adult ovarian cortex cultures (Li T et al. Unpublished). Finally, *Akr1b7*, *which* is involved in lipid detoxification process (Volle et al. 2004), is a major protein of the vas deferens in rodents (Baumann et al. 2007). Together, *Kcne2*, and to a lesser extent *Dhcr7* and *Akr1b7*, appear to be robust candidate biomarkers of ED exposure, being all upregulated after exposure to DES and KTZ, while *Inls3* was robustly under-expressed after exposure to these drugs, whatever the age analyzed.

## Conclusion

We previously showed that developmental exposure to DES and KTZ could induce expected endocrine-disrupting effects in exposed dams and male offspring, but not in female offspring. We surmised that this lack of effect outcomes had as much to do with the measurements being insensitive to detecting endocrine disruption in rodent toxicity studies as with the chemicals not affecting reproductive parameters in female offspring. By combining large-scale transcriptomic screening using BRB-seq with a deconvolution approach employing scRNA-seq datasets to discriminate bona fide changes to gene transcription from changes in ovary cellularity, we found the perinatal rat ovaries (PND 6-22) to be sensitive to perturbation by both DES and KTZ. We identified a suit of potential biomarkers of ovarian dysgenesis, some of which were common between the two chemicals. Many of the genes should be scrutinized further for their potential utility as biomarkers, both in combination and singularly. In particular, we consider *Kcne2*, *Calb2* and *Insl3* as highly interesting genes to investigate in additional rodent toxicity studies testing endocrine disruptors for potential impacts on the developing ovaries.

## Supplementary Information

Below is the link to the electronic supplementary material.

Supplementary file1 Workflow of the bulk (A) and single-cell (B) RNA-seq analyses. A) Flowchart of the methods used for the bulk RNA-seq analysis. Blue boxes represent data format. Grey boxes represent methods used to pass each format. B) Flowchart of the methods used for the single-cell RNA-seq analysis. Blue boxes represent data format. Grey boxes represent methods used to pass each format (PDF 84 KB)

Supplementary file2 Mouse ovary single-cell atlas data. A) Representation of the number of detected cells per sample (left) and the median number of detected genes per cell for each sample (right). The color gradient increases with the developmental stage. B) Distribution of developmental stages (left) and studies (right) per cluster. C) Spot plot representation of the expression of cell type markers across single-cell clusters. The size of a dot represents the percentage of cells in which a specific gene was detected for a given cluster, while its color represents the scaled expression value, according to the scale bars. D) UMAP representation of the expression of cell type markers. C/S = Coelomic/Surface; SG = Steroidogenic granulosa (PDF 1693 KB)

Supplementary file3 Deconvolution analysis of control samples. Boxplots showing predicted proportions of the eight most prominent ovarian cell types after deconvolution of bulk RNA-seq data. Control samples were from the plate containing DES exposed samples (Plate #2). Statistical comparisons were performed using the Wilcoxon rank-sum test, with asterisks indicating statistical significance. ns = non significatif; *p ≤ 0.05; **p ≤ 0.01; ***p ≤ 0.001. Coelomic/Surface epithelial cells (C/S epit. cells), Interstitial cells (Interst. cells), Granulosa cells (Gran. cells), Steroidogenic granulosa cells (SG cells), Endothelial cells (Endo. cells) (PDF 55 KB)

Supplementary file4 Deconvolution analysis on Diethylstilbestrol (DES) exposed samples. Boxplots showing predicted proportions of the eight most prominent ovarian cell types after exposure to DES at PND6, PND14 and PND22. Statistical comparisons were performed on control samples vs low, medium or high doses using Wilcoxon rank-sum test and the asterisks indicate statistical significance. ns = non significatif; *p ≤ 0.05; **p ≤ 0.01; ***p ≤ 0.001 (PDF 103 KB)

Supplementary file5 Deconvolution analysis on Ketoconazole (KTZ) exposed samples. Boxplots showing predicted cell proportions of the eight most prominent ovarian cell types after exposure to KTZ at PND6, PND14 and PND22. Statistical comparisons were performed on control samples vs low, medium or high doses using Wilcoxon rank-sum test and the asterisks indicate statistical significance. ns = non significatif; *p ≤ 0.05; **p ≤ 0.01; ***p ≤ 0.001 (PDF 97 KB)

Supplementary file6 Ovarian steroidogenesis pathway significantly affected by DES and KTZ. The molecules studied are represented in two columns for each gene (DES on the left and KTZ on the right), so that we can follow the impact of the molecules on the expression of genes involved in ovarian steroidogenesis. Genes significantly affected by DES are indicated with an orange circle, while those affected by KTZ are indicated with a green circle. We chose to show the general impact of the molecules regardless of the developmental stage or dose. The color-code indicates log2 fold-change value (PDF 126 KB)

Supplementary file7 Representations of scaled single-cell average expression values of the common DEGs. A) Spot plot representation of scaled single-cell average expression values in each cluster of the common DEGs. The size of a dot represents the percentage of cells in which a specific gene was detected for a given cluster, while its color represents the scaled expression value, according to the scale bars. Only genes retrieved in the single-cell study are represented. Those that were also found as cell type markers are indicated with asterisks. B) UMAP representation of scaled single-cell average expression values of the common DEGs. The color represents the scaled expression value, according to the scale bars in (A). Only genes retrieved in the single-cell study are represented. Those that were also found as cell type markers are indicated with asterisks (PDF 15338 KB)

Supplementary file8 (XLSX 494 KB)

Supplementary file9 (XLSX 110 KB)

Supplementary file10 (XLSX 2228 KB)

## Data Availability

Raw and preprocessed data were deposited at the GEO repository under the accession number GSE208545, and the transcriptomic signatures were deposited at the TOXsIgN repository under the accession number TSP1269 (https://toxsign.genouest.org/).

## References

[CR1] Abbott GW (2012). KCNE2 and the K (+) channel: the tail wagging the dog. Channels.

[CR2] Alpern D, Gardeux V, Russeil J, Mangeat B, Meireles-Filho ACA, Breysse R, Hacker D, Deplancke B (2019). BRB-seq: ultra-affordable high-throughput transcriptomics enabled by bulk RNA barcoding and sequencing. Genome Biol.

[CR3] Altobelli GG, Pentimalli F, D’Armiento M, Van Noorden S, Cimini V (2017). Calretinin immunoreactivity in the human testis throughout fetal life. J Cell Physiol.

[CR4] Baumann C, Davies B, Peters M, Kaufmann-Reiche U, Lessl M, Theuring F (2007). AKR1B7 (mouse vas deferens protein) is dispensable for mouse development and reproductive success. Reproduction.

[CR5] Benjamini Y, Hochberg Y (1995). Controlling the false discovery rate: a practical and powerful approach to multiple testing. J Roy Stat Soc: Ser B (methodol).

[CR6] Bertschy S, Genton CY, Gotzos V (1998). Selective immunocytochemical localisation of calretinin in the human ovary. Histochem Cell Biol.

[CR7] Buck Louis GM, Cooney MA, Peterson CM (2011). The ovarian dysgenesis syndrome. J Dev Orig Health Dis.

[CR8] Cardoso-Moreira M, Halbert J, Valloton D, Velten B, Chen C, Shao Y, Liechti A, Ascenção K, Rummel C, Ovchinnikova S, Mazin PV, Xenarios I, Harshman K, Mort M, Cooper DN, Sandi C, Soares MJ, Ferreira PG, Afonso S, Carneiro M, Turner JMA, VandeBerg JL, Fallahshahroudi A, Jensen P, Behr R, Lisgo S, Lindsay S, Khaitovich P, Huber W, Baker J, Anders S, Zhang YE, Kaessmann H (2019). Gene expression across mammalian organ development. Nature.

[CR9] Chalmel F, Primig M (2008). The annotation, mapping, expression and network (AMEN) suite of tools for molecular systems biology. BMC Bioinformatics.

[CR10] Chartrain I, Magre S, Maingourd M, Jost A (1984). Effect of serum on organogenesis of the rat testis in vitro. In Vitro.

[CR11] Darde TA, Gaudriault P, Beranger R, Lancien C, Caillarec-Joly A, Sallou O, Bonvallot N, Chevrier C, Mazaud-Guittot S, Jégou B, Collin O, Becker E, Rolland AD, Chalmel F (2018). TOXsIgN: a cross-species repository for toxicogenomic signatures. Bioinformatics.

[CR12] Draskau MK, Lardenois A, Evrard B, Boberg J, Chalmel F, Svingen T (2021). Transcriptome analysis of fetal rat testis following intrauterine exposure to the azole fungicides triticonazole and flusilazole reveals subtle changes despite adverse endocrine effects. Chemosphere.

[CR13] Draskau MK, Schwartz CL, Evrard B, Lardenois A, Pask A, Chalmel F, Svingen T (2022). The anti-androgenic fungicide triticonazole induces region-specific transcriptional changes in the developing rat perineum and phallus. Chemosphere.

[CR14] Edgar R, Domrachev M, Lash AE (2002). Gene expression omnibus: NCBI gene expression and hybridization array data repository. Nucleic Acids Res.

[CR15] Fisher CR, Graves KH, Parlow AF, Simpson ER (1998). Characterization of mice deficient in aromatase (ArKO) because of targeted disruption of the cyp19 gene. Proc Natl Acad Sci U S A.

[CR16] François CM, Petit F, Giton F, Gougeon A, Ravel C, Magre S, Cohen-Tannoudji J, Guigon CJ (2017). A novel action of follicle-stimulating hormone in the ovary promotes estradiol production without inducing excessive follicular growth before puberty. Sci Rep.

[CR17] Gal M, Eldar-Geva T, Margalioth EJ, Barr I, Orly J, Diamant YZ (1999). Attenuation of ovarian response by low-dose ketoconazole during superovulation in patients with polycystic ovary syndrome. Fertil Steril.

[CR18] Giacosa S, Pillet C, Séraudie I, Guyon L, Wallez Y, Roelants C, Battail C, Evrard B, Chalmel F, Barette C, Soleilhac E, Fauvarque M-O, Franquet Q, Sarrazin C, Peilleron N, Fiard G, Long J-A, Descotes J-L, Cochet C, Filhol O (2021). Cooperative blockade of CK2 and ATM kinases drives apoptosis in VHL-deficient renal carcinoma cells through ROS overproduction. Cancers.

[CR19] Guigon CJ, Mazaud S, Forest MG, Brailly-Tabard S, Coudouel N, Magre S (2003). Unaltered development of the initial follicular waves and normal pubertal onset in female rats after neonatal deletion of the follicular reserve. Endocrinology.

[CR20] Hao Y, Hao S, Andersen-Nissen E, Mauck WM, Zheng S, Butler A, Lee MJ, Wilk AJ, Darby C, Zager M, Hoffman P, Stoeckius M, Papalexi E, Mimitou EP, Jain J, Srivastava A, Stuart T, Fleming LM, Yeung B, Rogers AJ, McElrath JM, Blish CA, Gottardo R, Smibert P, Satija R (2021). Integrated analysis of multimodal single-cell data. Cell.

[CR21] Hatch EE, Troisi R, Wise LA, Hyer M, Palmer JR, Titus-Ernstoff L, Strohsnitter W, Kaufman R, Adam E, Noller KL, Herbst AL, Robboy S, Hartge P, Hoover RN (2006). Age at natural menopause in women exposed to diethylstilbestrol in utero. Am J Epidemiol.

[CR22] Hoover RN, Hyer M, Pfeiffer RM, Adam E, Bond B, Cheville AL, Colton T, Hartge P, Hatch EE, Herbst AL, Karlan BY, Kaufman R, Noller KL, Palmer JR, Robboy SJ, Saal RC, Strohsnitter W, Titus-Ernstoff L, Troisi R (2011). Adverse health outcomes in women exposed in utero to diethylstilbestrol. N Engl J Med.

[CR23] Howe KL, Achuthan P, Allen J, Allen J, Alvarez-Jarreta J, Amode MR, Armean IM, Azov AG, Bennett R, Bhai J, Billis K, Boddu S, Charkhchi M, Cummins C, Da Rin Fioretto L, Davidson C, Dodiya K, El Houdaigui B, Fatima R, Gall A, Garcia Giron C, Grego T, Guijarro-Clarke C, Haggerty L, Hemrom A, Hourlier T, Izuogu OG, Juettemann T, Kaikala V, Kay M, Lavidas I, Le T, Lemos D, Gonzalez Martinez J, Marugán JC, Maurel T, McMahon AC, Mohanan S, Moore B, Muffato M, Oheh DN, Paraschas D, Parker A, Parton A, Prosovetskaia I, Sakthivel MP, Salam AIA, Schmitt BM, Schuilenburg H, Sheppard D, Steed E, Szpak M, Szuba M, Taylor K, Thormann A, Threadgold G, Walts B, Winterbottom A, Chakiachvili M, Chaubal A, De Silva N, Flint B, Frankish A, Hunt SE, Iisley GR, Langridge N, Loveland JE, Martin FJ, Mudge JM, Morales J, Perry E, Ruffier M, Tate J, Thybert D, Trevanion SJ, Cunningham F, Yates AD, Zerbino DR, Flicek P (2021). Ensembl 2021. Nucleic Acids Res.

[CR24] Johansson HKL, Svingen T, Fowler PA, Vinggaard AM, Boberg J (2017). Environmental influences on ovarian dysgenesis–developmental windows sensitive to chemical exposures. Nat Rev Endocrinol.

[CR25] Johansson HKL, Svingen T, Boberg J, Fowler PA, Stead D, Vinggaard AM, Filis P (2020). Calretinin is a novel candidate marker for adverse ovarian effects of early life exposure to mixtures of endocrine disruptors in the rat. Arch Toxicol.

[CR26] Johansson HKL, Christiansen S, Draskau MK, Svingen T, Boberg J (2021). Classical toxicity endpoints in female rats are insensitive to the human endocrine disruptors diethylstilbestrol and ketoconazole. Reprod Toxicol.

[CR27] Kim J-M, Song K-S, Xu B, Wang T (2020). Role of potassium channels in female reproductive system. Obstet Gynecol Sci.

[CR28] Kjærstad MB, Taxvig C, Nellemann C, Vinggaard AM, Andersen HR (2010). Endocrine disrupting effects in vitro of conazole antifungals used as pesticides and pharmaceuticals. Reprod Toxicol.

[CR29] Korsunsky I, Nathan A, Millard N, Raychaudhuri S (2019). Presto scales Wilcoxon and auROC analyses to millions of observations. Biorxiv.

[CR30] Kundu P, Ciobotaru A, Foroughi S, Toro L, Stefani E, Eghbali M (2008). Hormonal regulation of cardiac KCNE2 gene expression. Mol Cell Endocrinol.

[CR31] Laronda MM, Unno K, Butler LM, Kurita T (2012). The development of cervical and vaginal adenosis as a result of diethylstilbestrol exposure in utero. Differentiation.

[CR32] Lê S, Josse J, Husson F (2008). Factominer: AnRPackage for multivariate analysis. J Stat Software.

[CR33] Leonard S, Lardenois A, Tarte K, Rolland AD, Chalmel F (2022). FlexDotPlot: a universal and modular dot plot visualization tool for complex multifaceted data. Bioinform Adv.

[CR34] Long X, Yang Q, Qian J, Yao H, Yan R, Cheng X, Zhang Q, Gu C, Gao F, Wang H, Zhang L, Guo F (2022). Obesity modulates cell-cell interactions during ovarian folliculogenesis. iScience.

[CR35] Love MI, Huber W, Anders S (2014). Moderated estimation of fold change and dispersion for RNA-seq data with DESeq2. Genome Biol.

[CR36] Lugli A, Forster Y, Haas P, Nocito A, Bucher C, Bissig H, Mirlacher M, Storz M, Mihatsch MJ, Sauter G (2003). Calretinin expression in human normal and neoplastic tissues: a tissue microarray analysis on 5233 tissue samples. Hum Pathol.

[CR37] Luo W, Brouwer C (2013). Pathview: an R/Bioconductor package for pathway-based data integration and visualization. Bioinformatics.

[CR38] Mack SO, Garrett WM, Guthrie HD (2000). Absence of correlation between in situ expression of cytochrome P450 17alpha hydroxylase/lyase and 3beta-hydroxysteroid dehydrogenase/(Delta5-4) isomerase messenger ribonucleic acids and steroidogenesis during pubertal development in the rat testis. J Steroid Biochem Mol Biol.

[CR39] Mason JI, Carr BR, Murry BA (1987). Imidazole antimycotics: selective inhibitors of steroid aromatization and progesterone hydroxylation. Steroids.

[CR40] Mazaud S, Guigon CJ, Lozach A, Coudouel N, Forest MG, Coffigny H, Magre S (2002). Establishment of the reproductive function and transient fertility of female rats lacking primordial follicle stock after fetal gamma-irradiation. Endocrinology.

[CR41] McGee EA, Hsu SY, Kaipia A, Hsueh AJ (1998). Cell death and survival during ovarian follicle development. Mol Cell Endocrinol.

[CR42] McGinnis CS, Murrow LM, Gartner ZJ (2019). DoubletFinder: doublet detection in single-cell RNA sequencing data using artificial nearest neighbors. Cell Syst.

[CR43] Meinsohn M-C, Saatcioglu HD, Wei L, Li Y, Horn H, Chauvin M, Kano M, Nguyen NMP, Nagykery N, Kashiwagi A, Samore WR, Wang D, Oliva E, Gao G, Morris ME, Donahoe PK, Pépin D (2021). Single-cell sequencing reveals suppressive transcriptional programs regulated by MIS/AMH in neonatal ovaries. Proc Natl Acad Sci USA.

[CR44] Munkboel CH, Rasmussen TB, Elgaard C, Olesen M-LK, Kretschmann AC, Styrishave B (2019). The classic azole antifungal drugs are highly potent endocrine disruptors in vitro inhibiting steroidogenic CYP enzymes at concentrations lower than therapeutic Cmax. Toxicology.

[CR45] Nakanishi T, Tanaka R, Tonai S, Lee JY, Yamaoka M, Kawai T, Okamoto A, Shimada M, Yamashita Y (2021). LH induces de novo cholesterol biosynthesis via SREBP activation in granulosa cells during ovulation in female mice. Endocrinology.

[CR46] Notarnicola C, Malki S, Berta P, Poulat F, Boizet-Bonhoure B (2006). Transient expression of SOX9 protein during follicular development in the adult mouse ovary. Gene Expr Patterns.

[CR47] OECD (2018). OECD Series on Testing and Assessment Revised Guidance Document 150 on Standardised Test Guidelines for Evaluating Chemicals for Endocrine Disruption.

[CR48] Palmer JR, Hatch EE, Rao RS, Kaufman RH, Herbst AL, Noller KL, Titus-Ernstoff L, Hoover RN (2001). Infertility among women exposed prenatally to diethylstilbestrol. Am J Epidemiol.

[CR49] Papanikolaou M, Crump SM, Abbott GW (2021). The focal adhesion protein Testin modulates KCNE2 potassium channel β subunit activity. Channels.

[CR50] Parsanezhad ME, Alborzi S, Pakniat M, Schmidt EH (2003). A double-blind, randomized, placebo-controlled study to assess the efficacy of ketoconazole for reducing the risk of ovarian hyperstimulation syndrome. Fertil Steril.

[CR51] Picut CA, Dixon D, Simons ML, Stump DG, Parker GA, Remick AK (2015). Postnatal ovary development in the rat: morphologic study and correlation of morphology to neuroendocrine parameters. Toxicol Pathol.

[CR52] Pohl V, Van Rampelbergh J, Mellaert S, Parmentier M, Pochet R (1992). Calretinin in rat ovary: an in situ hybridization and immunohistochemical study. Biochim Biophys Acta.

[CR53] Scarchilli L, Camaioni A, Bottazzi B, Negri V, Doni A, Deban L, Bastone A, Salvatori G, Mantovani A, Siracusa G, Salustri A (2007). PTX3 interacts with inter-alpha-trypsin inhibitor: implications for hyaluronan organization and cumulus oophorus expansion. J Biol Chem.

[CR54] Schwaller B (2014). Calretinin: from a “simple” Ca(2+) buffer to a multifunctional protein implicated in many biological processes. Front Neuroanat.

[CR55] Shin Y-H, McGuire MM, Rajkovic A (2013). Mouse HORMAD1 is a meiosis i checkpoint protein that modulates DNA double-strand break repair during female meiosis. Biol Reprod.

[CR56] Skakkebaek NE (2017). Sperm counts, testicular cancers, and the environment. BMJ.

[CR57] Smyth GK (2004). Linear models and empirical Bayes methods for assessing differential expression in microarray experiments. Stat Appl Genet Mol Biol.

[CR58] Steiner AZ, D’Aloisio AA, DeRoo LA, Sandler DP, Baird DD (2010). Association of intrauterine and early-life exposures with age at menopause in the Sister Study. Am J Epidemiol.

[CR59] Strauss KI, Isaacs KR, Ha QN, Jacobowitz DM (1994). Calretinin is expressed in the Leydig cells of rat testis. Biochim Biophys Acta.

[CR60] van Duursen MBM, Boberg J, Christiansen S, Connolly L, Damdimopoulou P, Filis P, Fowler PA, Gadella BM, Holte J, Jääger K, Johansson HKL, Li T, Mazaud-Guittot S, Parent A-S, Salumets A, Soto AM, Svingen T, Velthut-Meikas A, Wedebye EB, Xie Y, van den Berg M (2020). Safeguarding female reproductive health against endocrine disrupting chemicals-The FREIA project. Int J Mol Sci.

[CR61] Volle DH, Repa JJ, Mazur A, Cummins CL, Val P, Henry-Berger J, Caira F, Veyssiere G, Mangelsdorf DJ, Lobaccaro J-MA (2004). Regulation of the aldo-keto reductase gene akr1b7 by the nuclear oxysterol receptor LXRalpha (liver X receptor-alpha) in the mouse intestine: putative role of LXRs in lipid detoxification processes. Mol Endocrinol.

[CR62] Wang X, Park J, Susztak K, Zhang NR, Li M (2019). Bulk tissue cell type deconvolution with multi-subject single-cell expression reference. Nat Commun.

[CR63] Wang J-J, Ge W, Zhai Q-Y, Liu J-C, Sun X-W, Liu W-X, Li L, Lei C-Z, Dyce PW, De Felici M, Shen W (2020). Single-cell transcriptome landscape of ovarian cells during primordial follicle assembly in mice. PLoS Biol.

[CR64] Xu W, Zhu Q, Liu S, Dai X, Zhang B, Gao C, Gao L, Liu J, Cui Y (2018). Calretinin participates in regulating steroidogenesis by PLC-Ca2+-PKC pathway in leydig cells. Sci Rep.

[CR65] Yazawa T, Imamichi Y, Sekiguchi T, Miyamoto K, Uwada J, Khan MRI, Suzuki N, Umezawa A, Taniguchi T (2019). Transcriptional regulation of ovarian steroidogenic genes: recent findings obtained from stem cell-derived steroidogenic cells. Biomed Res Int.

[CR66] Zhang FP, Poutanen M, Wilbertz J, Huhtaniemi I (2001). Normal prenatal but arrested postnatal sexual development of luteinizing hormone receptor knockout (LuRKO) mice. Mol Endocrinol.

[CR67] Zimmermann S, Steding G, Emmen JM, Brinkmann AO, Nayernia K, Holstein AF, Engel W, Adham IM (1999). Targeted disruption of the Insl3 gene causes bilateral cryptorchidism. Mol Endocrinol.

