# Supplementary figures and images for: Transcriptional profiling of the developing rat ovary following intrauterine exposure to the endocrine disruptors diethylstilbestrol and ketoconazole
Source: Arch Toxicol. 2023 Jan 18;97(3):849–63. doi: 10.1007/s00204-023-03442-2 (PMC9968686; doi:10.1007/s00204-023-03442-2)

**A****Bulk RNA-seq analysis**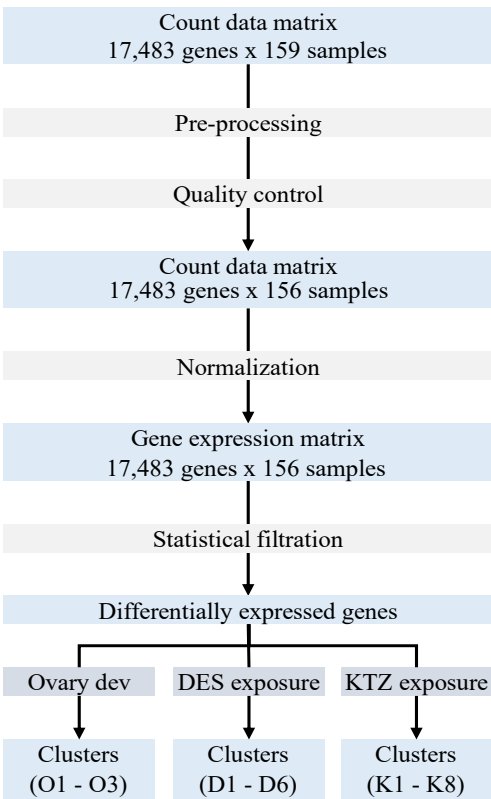**B****Single-cell RNA-seq analysis**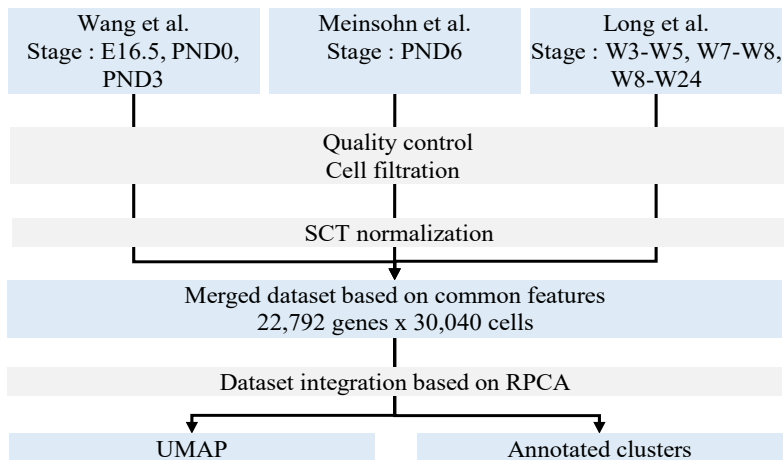**Figure S1**

Supplement: Supplementary file 1 — Supplementary file1 Workflow of the bulk (A) and single-cell (B) RNA-seq analyses. A) Flowchart of the methods used for the bulk RNA-seq analysis. Blue boxes represent data format. Grey boxes represent methods used to pass each format. B) Flowchart of the methods used for the single-cell RNA-seq analysis. Blue boxes represent data format. Grey boxes represent methods used to pass each format (PDF 84 KB) [file 204_2023_3442_MOESM1_ESM.pdf]

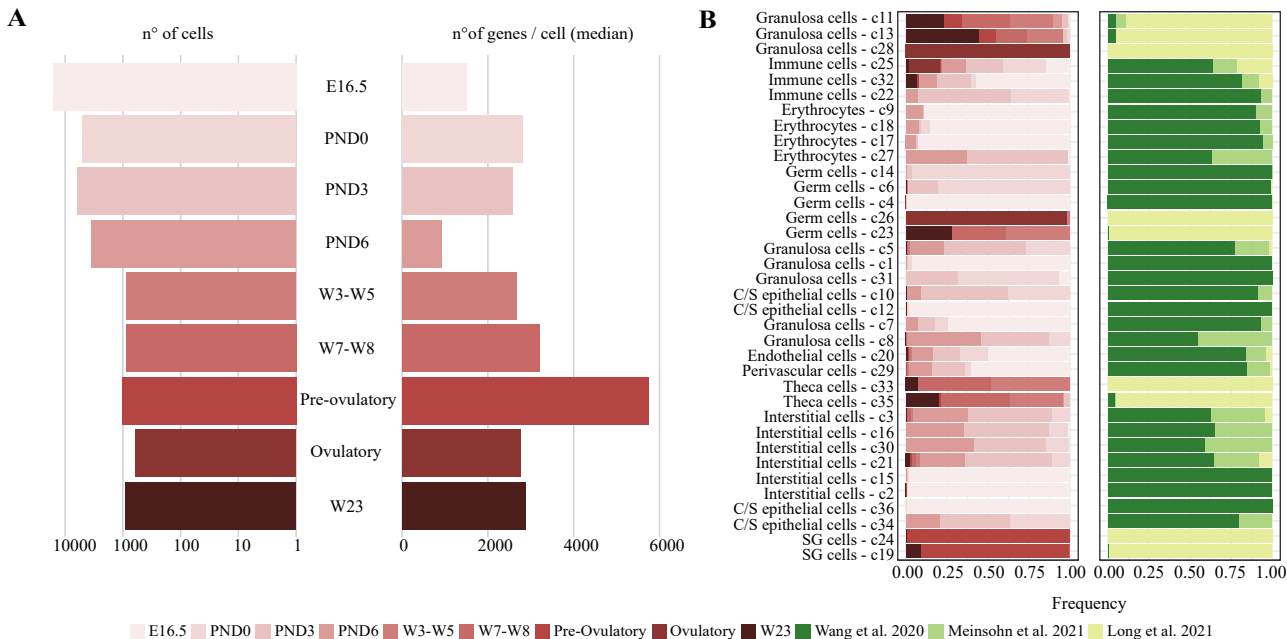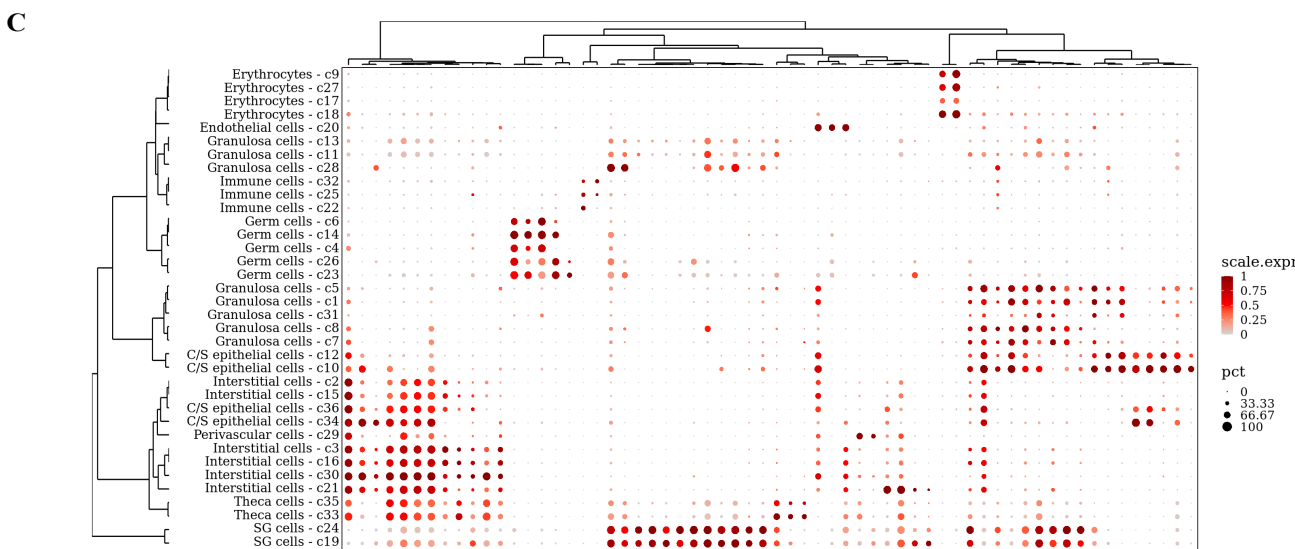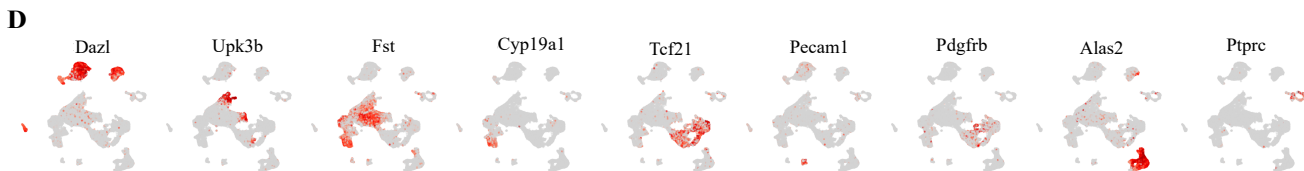

**Figure S2**

Supplement: Supplementary file 2 — Supplementary file2 Mouse ovary single-cell atlas data. A) Representation of the number of detected cells per sample (left) and the median number of detected genes per cell for each sample (right). The color gradient increases with the developmental stage. B) Distribution of developmental stages (left) and studies (right) per cluster. C) Spot plot representation of the expression of cell type markers across single-cell clusters. The size of a dot represents the percentage of cells in which a specific gene was detected for a given cluster, while its color represents the scaled expression value, according to the scale bars. D) UMAP representation of the expression of cell type markers. C/S = Coelomic/Surface; SG = Steroidogenic granulosa (PDF 1693 KB) [file 204_2023_3442_MOESM2_ESM.pdf]

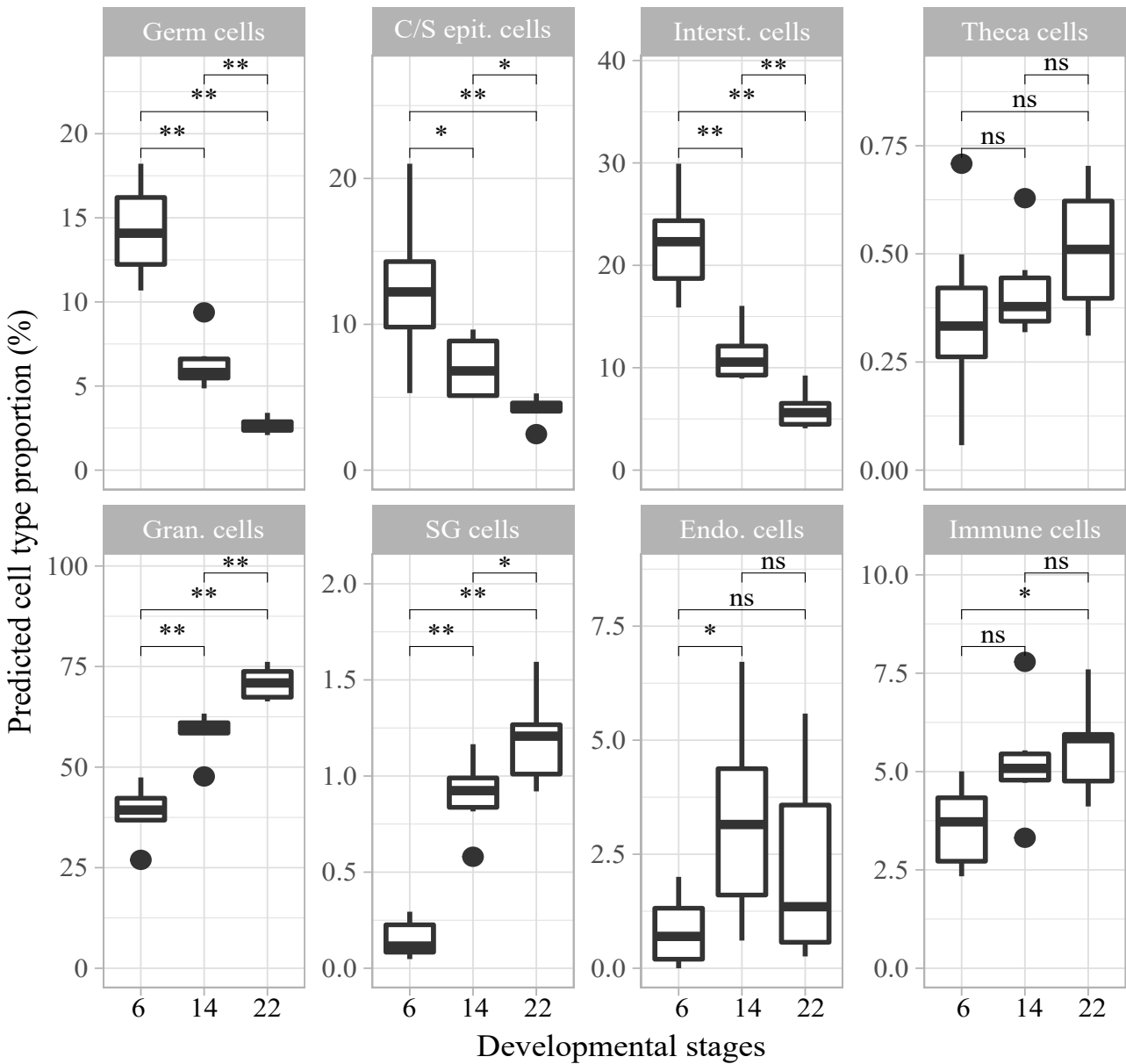

**Figure S3**

Supplement: Supplementary file 3 — Supplementary file3 Deconvolution analysis of control samples. Boxplots showing predicted proportions of the eight most prominent ovarian cell types after deconvolution of bulk RNA-seq data. Control samples were from the plate containing DES exposed samples (Plate #2). Statistical comparisons were performed using the Wilcoxon rank-sum test, with asterisks indicating statistical significance. ns = non significatif; *p ≤ 0.05; **p ≤ 0.01; ***p ≤ 0.001. Coelomic/Surface epithelial cells (C/S epit. cells), Interstitial cells (Interst. cells), Granulosa cells (Gran. cells), Steroidogenic granulosa cells (SG cells), Endothelial cells (Endo. cells) (PDF 55 KB) [file 204_2023_3442_MOESM3_ESM.pdf]

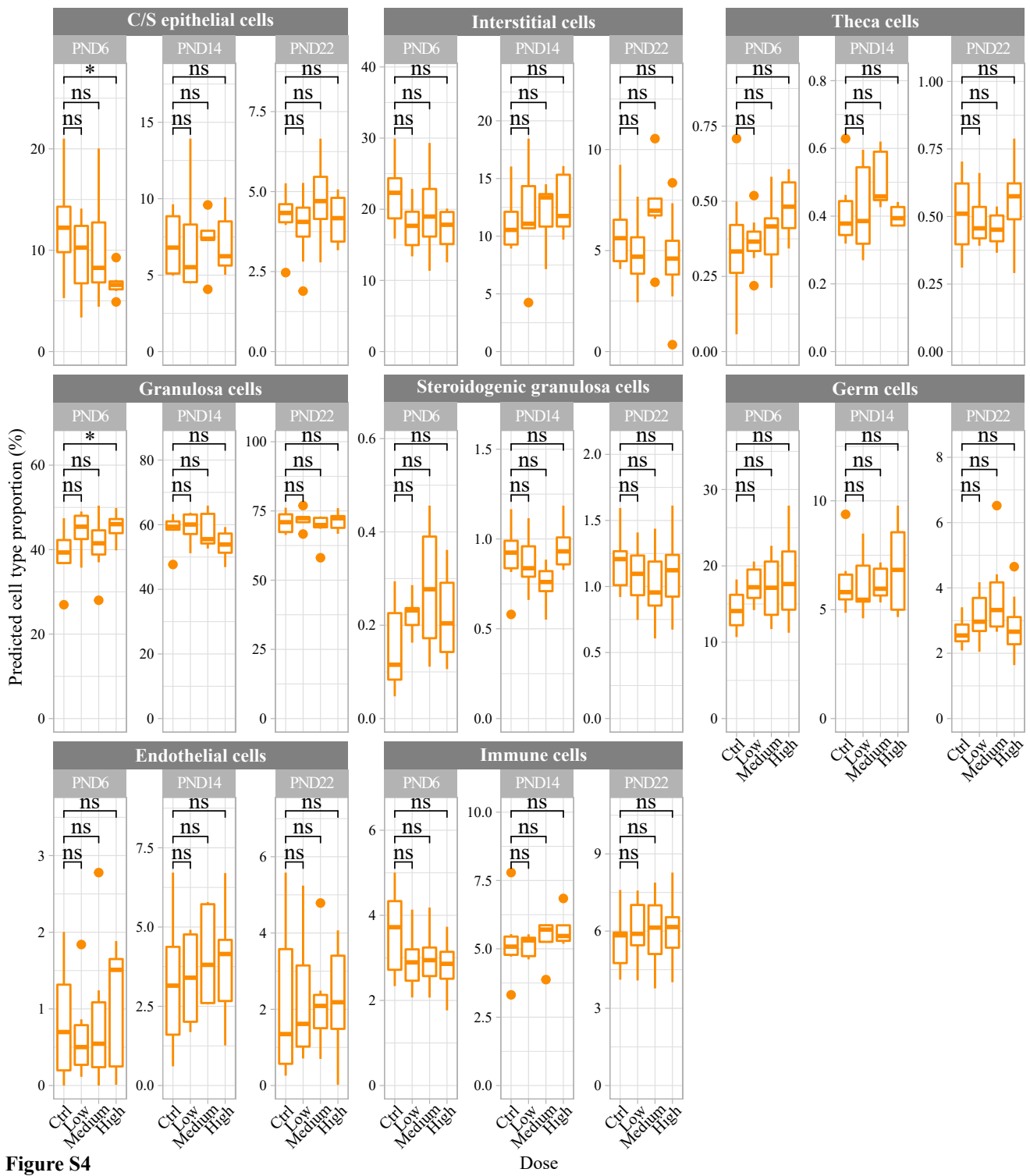

Supplement: Supplementary file 4 — Supplementary file4 Deconvolution analysis on Diethylstilbestrol (DES) exposed samples. Boxplots showing predicted proportions of the eight most prominent ovarian cell types after exposure to DES at PND6, PND14 and PND22. Statistical comparisons were performed on control samples vs low, medium or high doses using Wilcoxon rank-sum test and the asterisks indicate statistical significance. ns = non significatif; *p ≤ 0.05; **p ≤ 0.01; ***p ≤ 0.001 (PDF 103 KB) [file 204_2023_3442_MOESM4_ESM.pdf]

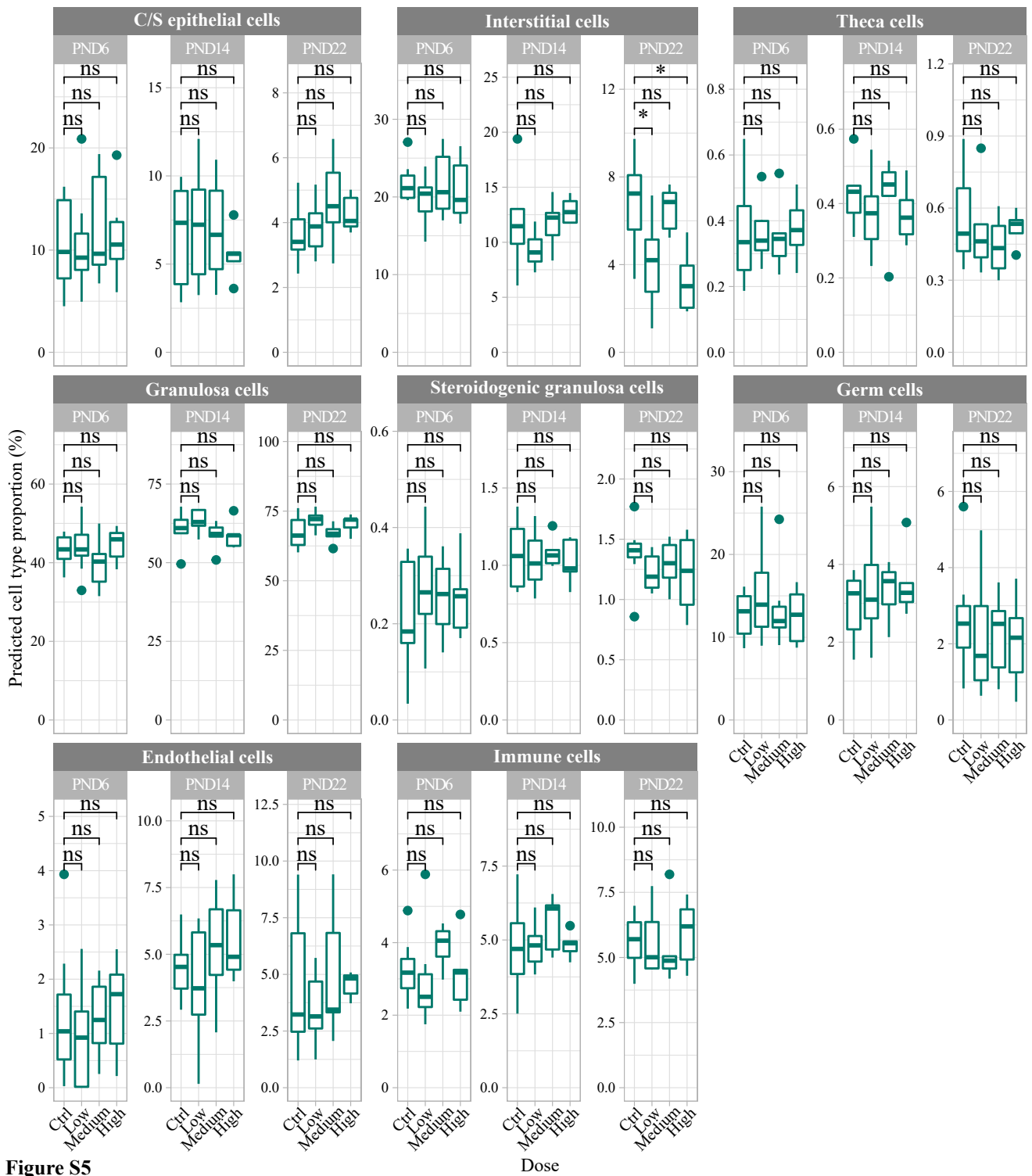

Supplement: Supplementary file 5 — Supplementary file5 Deconvolution analysis on Ketoconazole (KTZ) exposed samples. Boxplots showing predicted cell proportions of the eight most prominent ovarian cell types after exposure to KTZ at PND6, PND14 and PND22. Statistical comparisons were performed on control samples vs low, medium or high doses using Wilcoxon rank-sum test and the asterisks indicate statistical significance. ns = non significatif; *p ≤ 0.05; **p ≤ 0.01; ***p ≤ 0.001 (PDF 97 KB) [file 204_2023_3442_MOESM5_ESM.pdf]

# OVARIAN STEROIDOGENESIS

Log2 Fold-change

-1 0 1

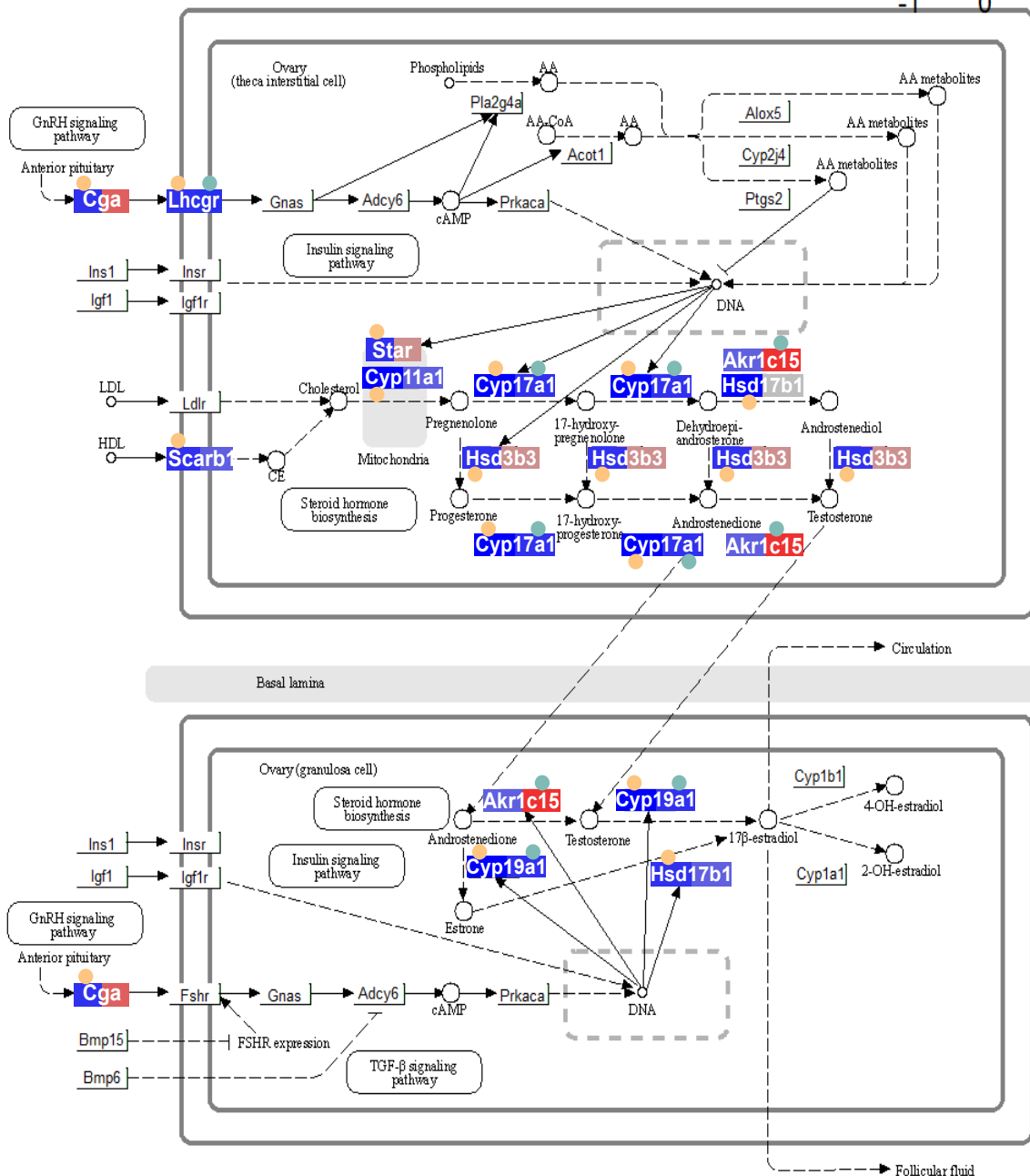

Data on KEGG graph  
Rendered by Pathview

Figure S6

Supplement: Supplementary file 6 — Supplementary file6 Ovarian steroidogenesis pathway significantly affected by DES and KTZ. The molecules studied are represented in two columns for each gene (DES on the left and KTZ on the right), so that we can follow the impact of the molecules on the expression of genes involved in ovarian steroidogenesis. Genes significantly affected by DES are indicated with an orange circle, while those affected by KTZ are indicated with a green circle. We chose to show the general impact of the molecules regardless of the developmental stage or dose. The color-code indicates log2 fold-change value (PDF 126 KB) [file 204_2023_3442_MOESM6_ESM.pdf]

A

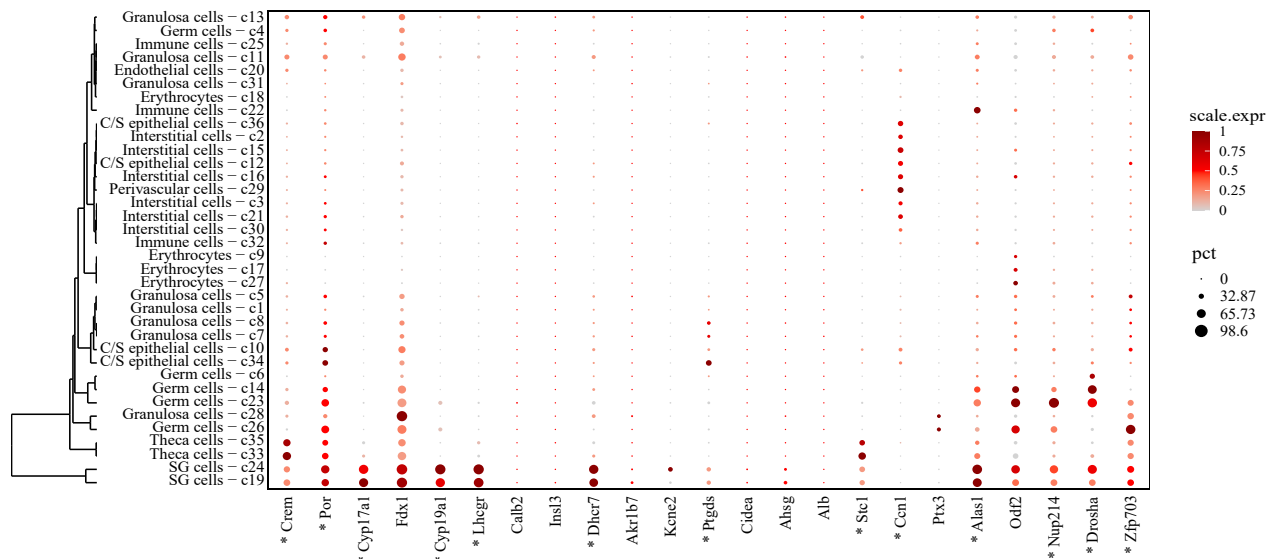

B

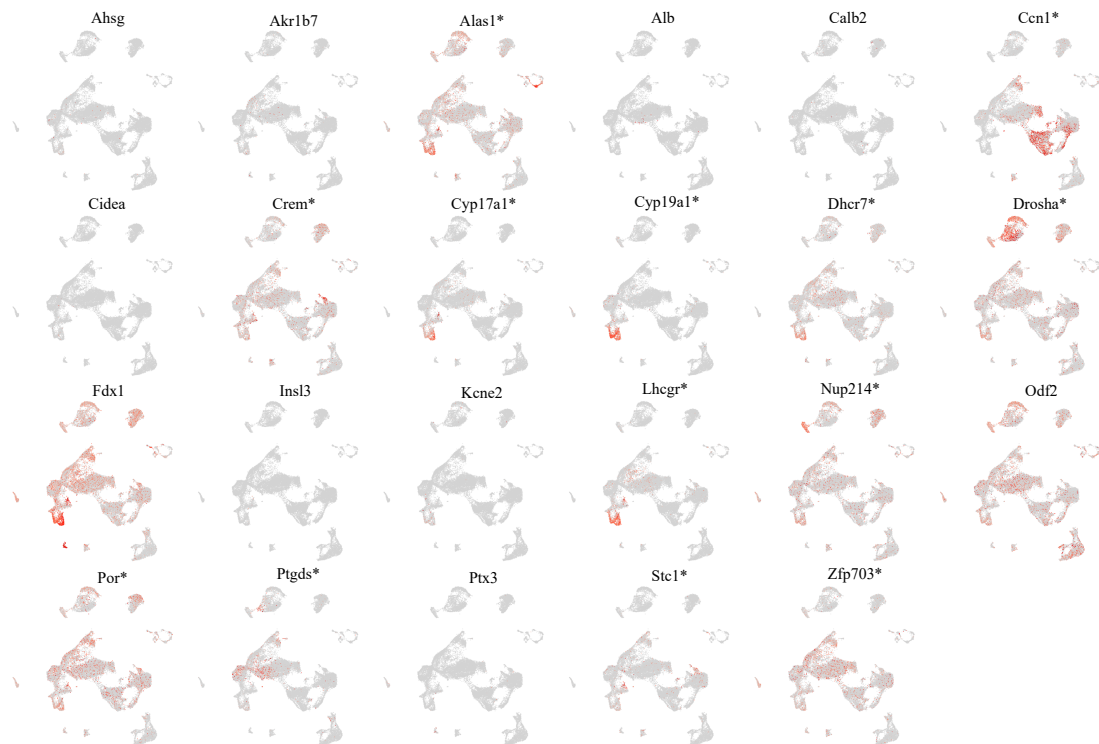

Figure S7

Supplement: Supplementary file 7 — Supplementary file7 Representations of scaled single-cell average expression values of the common DEGs. A) Spot plot representation of scaled single-cell average expression values in each cluster of the common DEGs. The size of a dot represents the percentage of cells in which a specific gene was detected for a given cluster, while its color represents the scaled expression value, according to the scale bars. Only genes retrieved in the single-cell study are represented. Those that were also found as cell type markers are indicated with asterisks. B) UMAP representation of scaled single-cell average expression values of the common DEGs. The color represents the scaled expression value, according to the scale bars in (A). Only genes retrieved in the single-cell study are represented. Those that were also found as cell type markers are indicated with asterisks (PDF 15338 KB) [file 204_2023_3442_MOESM7_ESM.pdf]
